# An extragenic fix for a broken enzyme

**DOI:** 10.1099/mic.0.001760

**Published:** 2026-10-05

**Authors:** Veena Mohan, Edoardo Labrini, Cemile Selin Aksoy, Volker Behrends, Laila C. Evangelista, David R. Spring, Martin Welch

**Affiliations:** 1 Department of Biochemistry (Hopkins Building), University of Cambridge, Tennis Court Road, Cambridge, CB2 1QW, UK; 2 Yusuf Hamied Department of Chemistry, University of Cambridge, Lensfield Road, Cambridge, CB2 1EW, UK; 3 Biomedical Device Technology Program, Vocational School of Health Services, Izmir Democracy University, 35290, Izmir, Turkey; 4 School of Medicine and Biosciences, University of West London, St. Marys Road, London, W5 5RF, UK; 5 Department of Surgery & Cancer, Institute of Reproductive Development Building, Hammersmith Hospital, Du Cane Road, London W12 0NN, UK

**Keywords:** arginine metabolism, auxotrophy, enzyme regulation, evolution, proteomics, *Pseudomonas aeruginosa*

## Abstract

In this work, we show how exploration of genomic sequence space yields an unexpected evolutionary ‘fix’ that allows the cell to bypass the phenotypic defects associated with a dysfunctional enzyme. We identified a transposon (Tn) insertion in the gene (*argA*) encoding *N*-acetylglutamate synthase (NAGS) that leads to arginine auxotrophy in *Pseudomonas aeruginosa*. This Tn insertion generates a truncated version of the ArgA protein (ArgA_188-432_) that retains NAGS activity but, unlike the wild-type protein, is unrestrained by arginine feedback inhibition. However, the Tn::*argA* mutant readily re-acquired a prototrophic phenotype through the acquisition of spontaneous secondary mutations outside of the *argA* locus. Whole-genome sequencing and complementation analyses of one such ‘bypass mutant’ allowed us to trace this epistatic effect to an Ala128→Val substitution in AmbA, a LysE-type transmembrane transporter. Using a variety of genetic approaches, we show that the *ambA*^Ala128Val^ mutation gives rise to a change/expansion of function in the encoded gene product and that this mutation is directly or indirectly responsible for bypassing the auxotrophy associated with the original Tn::*argA* mutation. Further analysis of the Tn::*argA ambA*^Ala128Val^ mutant revealed that, compared with the ‘wild-type’ progenitor, the mutant exhibited large-scale changes in its proteome and metabolome consistent with elevated intracellular arginine and enhanced polyamine degradation. We conclude that the *ambA*^Ala128Val^ mutation overcomes the phenotypic impact of the dysregulated ArgA_188-432_ enzyme, possibly by enabling efflux of toxic polyamines (which we also show can competitively inhibit NAGS).

## Data Availability

The proteomics data have been deposited on PRIDE with the accession number PXD075063. The Whole Genome Shotgun data have been deposited at DDBJ/ENA/GenBank under the accession JBZCIP010000000. The metabolomics data have been deposited at the NIH Metabolomics Workbench with accession (project) number ST004923.

## Introduction

*Pseudomonas aeruginosa* (PA) is a Gram-negative, opportunistic pathogen capable of causing life-threatening infections. Although commonly cited as being ‘ubiquitous’, the organism appears to exhibit a proclivity for colonizing niches in the built environment and/or places associated with human activity [1]. Consequently, and for persons predisposed towards infection by this organism, it can be difficult to not eventually acquire PA. For example, the airways of people with cystic fibrosis (CF) appear to be remarkably susceptible towards infection by PA, such that by their late teens, many people with CF harbour chronic PA infections [2]. These chronic PA infections used to be thought of as being ‘seeded’ by cells from environmental reservoirs, although more recent molecular-genetic data indicate that many CF-associated strains are acquired by cross-infection between patients [3], with some so-called ‘epidemic strains’ showing a particular predilection for infecting CF hosts [4]. Interestingly, there appears to be a correlation between the transmissibility of infecting strains and their ability to produce virulence factors such as elastase [5].

Although usually clonally derived, the PA population in a CF airway is frequently heterogeneous and can be thought of as being a mixture of co-habiting relatives. These variants often exhibit diverse phenotypic traits such as altered expression of virulence factors, increased mucoidy, elevated resistance to antimicrobial agents, hypermutability and decreased motility [6]. Whole-genome sequencing (WGS) efforts have shown that the acquisition of these traits is underpinned by the appearance of SNPs and indels in a relatively well-defined set of genes [7–9]. The significance of these changes has been extensively discussed in the literature over the last decade, with particular attention being paid to SNPs affecting global virulence regulatory pathways such as quorum sensing. However, perhaps less well investigated are the more prosaic phenotypes, such as amino acid auxotrophy, even though auxotrophic PA isolates were identified very early on as being a common feature in expectorated CF sputum [10]. The most frequently isolated PA auxotrophs are defective in the biosynthesis of methionine, branched chain amino acids (Leu, Val, Ile) or arginine and therefore require these amino acids to be present in the medium for growth [11, 12]. Quite why these particular amino acid biosynthetic pathways are susceptible to preferential loss of function is not clear, although PA does exhibit a strong preference for consuming methionine over most other amino acids. By contrast, arginine and leucine are ‘middle favoured’, whereas valine is among the least readily consumed [13]. Mitigating, we note that PA metabolism displays pronounced reverse diauxie, meaning that under some conditions the organism preferentially metabolizes substrates that do not maximize growth rate. Furthermore, adaptation to the CF airway environment leads to longitudinal changes in amino acid preference [14].

The initial driver behind the work described here was to characterize an arginine auxotroph better, with a view to identifying possible associated phenotypes that might confer a fitness advantage. We chose to look at arginine because this amino acid has been implicated in promoting virulence in infection scenarios [15, 16]. The arginine biosynthetic genes of PA are distributed across the genome as monocistronic units. The first enzyme in the pathway is *N*-acetylglutamate synthase (NAGS). In PA, arginine biosynthesis proceeds via the transfer of an acetyl moiety from acetyl-CoA to l-glutamate, forming *N*-acetyl l-glutamate (NAG). This reaction is catalysed by the enzyme NAGS (Fig. 1). The *N*-acetylation reaction is important because it prevents spontaneous cyclization of the glutamate derivative, which could otherwise lead to the formation of unwanted proline-like cyclicals. The synthesis of arginine subsequently follows a linear pathway that eventually leads to the formation of the ornithine precursor, *N*-acetyl ornithine. This product has two fates. It may be deacetylated by ArgE to yield ornithine and free acetate, or the activated acetyl group can be recycled to acetylate l-glutamate in a reaction catalysed by ArgJ (ornithine acetyltransferase, OAT). In many organisms, the OAT-catalysed reaction has been proposed as the main source of NAG and NAGS has been suggested to have a primarily anaplerotic role [17]. However, in PA, mutants defective in NAGS activity have been reported to be arginine auxotrophs [18, 19], whereas mutants defective in *argJ* are not [20]. Similarly, disruption of the *argA* ORF in a closely related species (*Pseudomonas syringae*) also leads to arginine auxotrophy [21]. Taken together, these data suggest that ArgA likely does more than simply ‘prime’ the pathway with an initial pulse of activated acetate; rather, it plays an indispensable role in arginine biosynthesis.

**Fig. 1.**
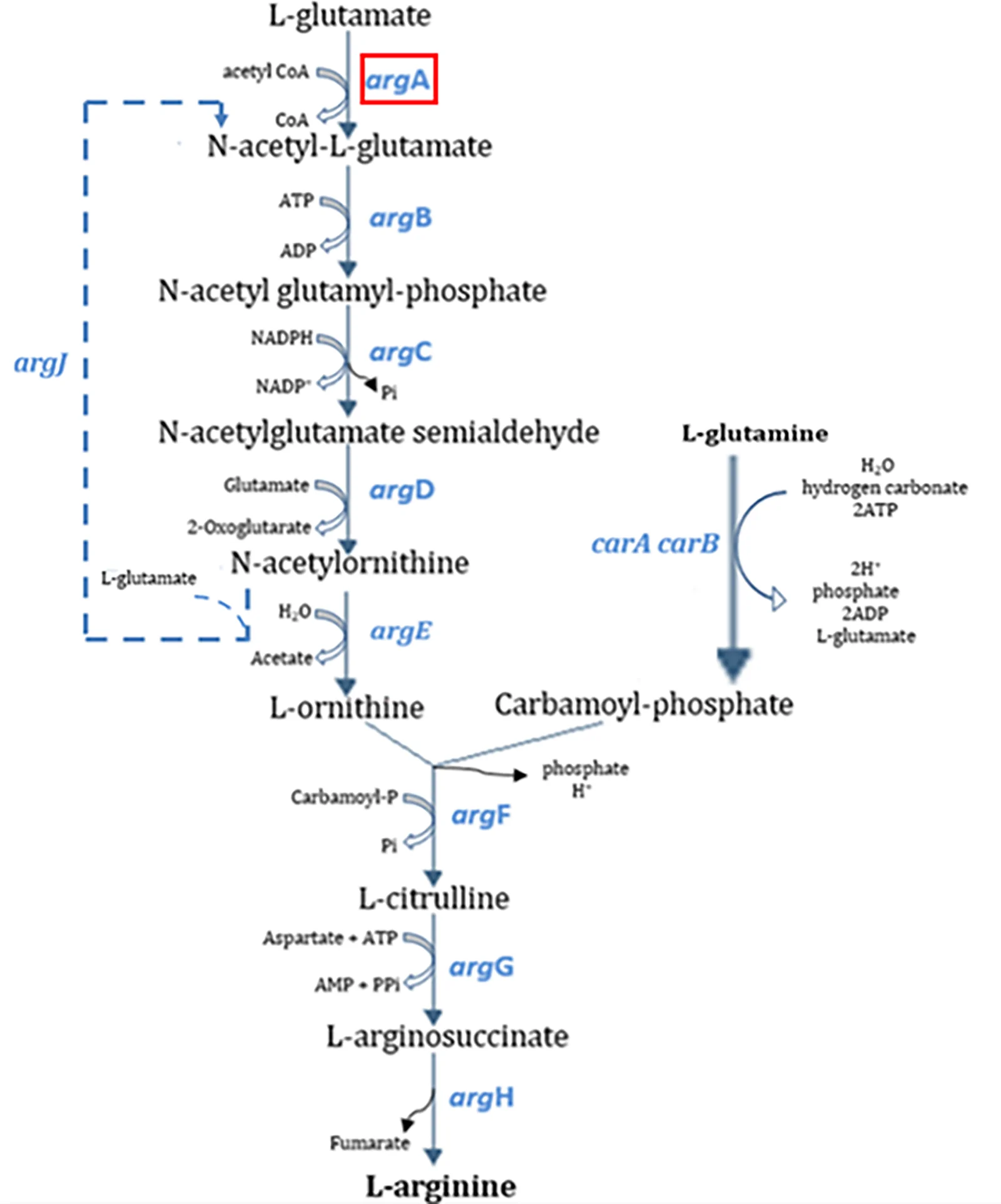
Arginine biosynthesis in PA. The biosynthetic genes *argA*, *argB*, *argC*, *argD*, *argE*, *argF*, *argG* and *argH* encode the enzymes NAGS, *N*-acetylglutamate kinase, *N*-acetyl γ-glutamyl-phosphate reductase, *N*-acetylornithine 5-amino transferase, acetyl ornithinase, ornithine carbamoyltransferase (anabolic), arginosuccinate synthase and arginosuccinate lyase, respectively. The ‘cyclic pathway’ (dashed blue line) for synthesizing *N*-acetyl glutamate from *N*-acetylornithine is catalysed by the OAT, ArgJ. Carbamoyl phosphate for l-citrulline synthesis is generated by the activity of carbamoyl phosphate synthase (encoded by *carA* and *carB*). The ArgA enzyme studied in the current work is boxed in red.

The NAGS activity of ArgA in PA is strongly inhibited by arginine [22]. In the current work, we identify a mutant of PAO1 that contains a transposon (Tn) insertion towards the 5′ end of *argA* in the arginine-binding domain of the encoded protein. This mutant expresses a catalytically active NAGS but is insensitive to arginine inhibition. Remarkably, and in spite of its robust NAGS activity, this mutant is an arginine auxotroph. This was surprising because as noted above, previous work by Haas and colleagues in the 1970s indicated that arginine auxotrophy is normally associated with complete loss of NAGS activity in PA [18], yet our data suggest that dysregulation of ArgA can also lead to auxotrophy. However, we found that the arginine auxotrophy in the dysregulated mutant could be readily ‘bypassed’ through the acquisition of secondary mutations elsewhere in the genome, leading to the restoration of prototrophy. This led us to investigate one such *argA* bypass mutation in detail. Unexpectedly, we found that the restoration of prototrophy in this mutant was driven by a change-of-function (CoF)/expansion-of-function (EoF) mutation in an anti-metabolite transporter, AmbA. The possible mechanism(s) by which this is accomplished are discussed.

## Methods

### Construction of complementing plasmids

The a*rgA and argF* ORFs were PCR-amplified from PAO1 genomic DNA (gDNA) using primers 5′-ATGCGGATCCGCTGCGACGACGATAACGGCGC-3′ and 5′- ATGCAAGCTTCCTGATCAGCAACTGAGCCAAGC-3′ (for *argA*) and 5′- GATCGGATCCACGTTGCGGTCCCGGCGCAGGTC-3′ and 5′- GATCAAGCTTCAGGTTCAGCAGCAGGTCGTGG-3′ (for *argF*). Following cleanup, the PCR products were digested with *Bam*HI and *Hind*III and ligated to similarly cleaved pUCP20 to yield pUCP20*argA* and pUCP20*argF*. The insertions were confirmed by Sanger sequencing.

### PCR amplification of the *argA* locus from strains PAO366 and PAO540

gDNA was extracted from overnight LB-grown cultures of the NAGS-deficient *argA* mutants, PAO366 and PAO540. This gDNA was used as a template to PCR amplify the *argA* ORF (including 70 bp upstream sequence and 144 bp downstream sequence) from each strain. The primers used were 5′-GCTGCGACGACGATAAC-3′ (upstream) and 5′-GAAGATGGCCAGCGAC-3′ (downstream), and the annealing temperature was 60 °C. The same primers were used to sequence the PCR products.

### Cloning of full-length and truncated *argA*

The ORF encoding *argA* was PCR-amplified with the primers 5′-tatgggacccggggtcgacctcgagATGCCCGACTACGTCAAC-3′ and 5′-tttgttagcagccggatccctcgagTCACAGGCTCTTCTCGAATAC-3′. The resulting amplicon was purified using the Monarch^®^ Spin PCR and DNA Cleanup Kit (NEB) and ligated into XhoI-digested pET-19m, which incorporates a TEV-cleavable N-terminal His_8_-tag. To clone and His-tag the region downstream of Met188, the primers 5′-tatgggacccggggtcgacctcgagATGCGCGCCGCCATCGAC-3′ and 5′-tttgttagcagccggatccctcgagTCACAGGCTCTTCTCGAATACCTGCG-3′ were used to amplify the relevant region of *argA*, which was subsequently ligated into XhoI-digested pET-19m. The NEBuilder^®^ HiFi DNA Assembly Cloning Kit (NEB) was used to clone each PCR fragment into pET-19m. Constructs were verified by Sanger sequencing.

### Confirmation of Tn insertion location in the *argA* mutant

The Tn insertion was confirmed by PCR-based amplification across the insertion sites. To PCR amplify across the expected Tn/*argA* junctions in PW9762 (the Tn::*argA* mutant), we used primer pairs 5′-AGAAGCCTGTCGATGTTGC-3′ (annealing just upstream of *argA*) and 5′-AGCTACTGGGCTATCTGGAC-3′ (reading out of the Tn) and 5′-AGAAGCCTGTCGATGTTGC-3′ (located within the *argA* sequence, but downstream of the probable Tn insertion site) and 5′-CGAGTCGGTGACGTAGTCC-3′ (reading out of the Tn). Following cleanup, the PCR products were confirmed by Sanger sequencing.

### Protein purification

For overexpression of N-terminally His_6_-tagged full-length ArgA (ArgA_1-432_) or truncated ArgA (ArgA_188-432_), Rosetta BL21 cells containing the relevant pET19m-derived recombinant plasmid were grown at 37 °C with good aeration (shaking at 200 r.p.m.) in 1 l of LB medium supplemented with carbenicillin (50 µg ml^−1^) and chloramphenicol (34 µg ml^−1^) to an OD_600_ of 0.5–0.6. The temperature was then lowered to 20 °C, and IPTG was added to the culture to a final concentration of 1 mM to induce expression of the cloned gene. The induced culture was incubated for a further 16 h and then harvested by centrifugation (3,430 ***g***, 20 min, 4 °C). The cell pellet was re-suspended in 20 ml of ice-cold lysis buffer [50 mM Tris/HCl (pH 8.0), 400 mM NaCl, 5% v/v glycerol, 10 mM imidazole] containing an EDTA-free protease inhibitor cocktail tablet (Sigma) and ruptured by sonication (10×30 s, 15 amps) on ice. The cell lysate was clarified by centrifugation (9,900 ***g***, 20 min, 4 °C) to remove cellular debris, and the supernatant was filtered through a 0.45 µm filter (Sartorius). The clarified supernatant was then loaded onto a nickel-nitrilotriacetic Acid (Ni-NTA) column (2 ml packed bed volume) pre-equilibrated with lysis buffer. Unless otherwise indicated, all protein purification and handling steps were carried out at 4 °C. After washing the column overnight (flow rate ~0.5 ml min^−1^) with ~200 column volumes of lysis buffer, the bound proteins were eluted in ~10 ml of 50 mM Tris/HCl (pH 8.0), 100 mM NaCl, 300 mM imidazole. The eluted protein was concentrated by ultrafiltration and flash frozen in aliquots. The concentration of the full length (47.8 kDa, ε=27,400) and truncated (27.2 kDa, ε=14,400) proteins were determined by spectrophotometry at 280 nm.

### NAGS assay

The standard assay mixture contained a solution of 18 µl of 0.2 M Tris/HCl (pH 9.0), 15 mM l-glutamate (sodium salt), 3 mM acetyl-CoA and where required, 2 mM l-arginine, as indicated. Fresh enzyme (typically 1 µl of ~1 mg ml^−1^ stock solution) was added to the mixture to initiate the reaction, and the samples were incubated at 37 °C. After a period of incubation (10–40 min, as indicated), 800 µl of 0.2 mM 5,5’-dithiobis(2-nitrobenzoic acid) (DTNB; Ellman’s reagent) was added. The DTNB was initially dissolved in 0.5 ml DMSO, and then immediately before quenching the reaction, was further diluted into 100 ml PBS (pH adjusted to pH 8.0). Colour production was confirmed to be linear over the reaction course. Reaction mixtures without enzyme were used as blank to correct for spontaneous AcCoA hydrolysis. Absorbance was measured at 412 nm to quantify CoA release using a Biospectrometer (Eppendorf).

### Whole-genome sequencing

The Tn::*argA* mutant (PW9762) and a selection of spontaneous ‘bypass’ mutants (restored for arginine prototrophy) were sent to MicrobesNG for WGS using an Illumina HiSeq 2500 platform. gDNA libraries were prepared using Nextera XT Library Prep Kit (Illumina) following the manufacturer’s protocol, and the libraries were sequenced using a 250 bp paired-end protocol. Reads were adapter-trimmed using Trimmomatic 0.30 [23] with a sliding window quality cut-off Q15. *De novo* assembly was performed using SPAdes version 3.7 [24], and contigs were annotated using Prokka 1.11 [25]. The Tn::*argA* mutant genome was first aligned with the progenitor WT genome (MPAO1; the WGS of the lab lineage is known) using the Multiple Genome Alignment tool, Mauve [26]. The aligned Tn::*argA* progenitor was then used as the reference genome to align the derivative Tn::*argA* (bypass) mutants. The FASTA files of each bypass mutant were mapped to the Tn::*argA* progenitor assembly using bwa version 0.7.17. SNPs were called from the resulting BAM files using samtools mpileup version 1.6 [27] and BCFtools call version 1.5. SNPs were called if 80% of reads for each isolate supported the SNP, with a minimum depth of 8×. Consensus sequences were called with samtools version 1.6 as FASTA files, annotated using PROKKA version 1.5 as GFF3 files, and aligned using Mauve version 2.4.0 into a multi-FASTA file. The ‘called SNPs’ were collected into a pseudo-VCF file using SNP-sites version 2.4.1 [28].

### Proteomics

Wild-type (MPAO1 and LPAO1) and mutants [Δ*ambA* and AMB128 (Tn::*argA ambA*)] were cultured to late exponential phase (OD_600_ of 0.7–0.8) in MOPS minimal media supplemented with 0.25% (wt/v) glucose. Cell cultures (45 ml) were harvested by pelleting at 3,200 ***g*** for 30 min at 4 °C, washed in PBS and sedimented a second time. Pellets were re-suspended in 800 µl of lysis buffer and sonicated (3×5 s at 15 amps, MSE microtip). Cells were pelleted at 21,000 ***g*** for 30 min at 4 °C. The protein concentration of the supernatant was determined using the DC protein assay (Bio-Rad).

The Cambridge Centre for Proteomics performed the tryptic digestion/reduction/alkylation of the samples, the LC–MS/MS-based quantitative proteomic analysis, and data processing, essentially as previously described [29]. Briefly, the dried peptides were reconstituted in 100 mM triethylammonium bicarbonate and labelled using 10-plex TMT reagents according to the manufacturer’s (Thermo Fisher Scientific) protocol. Tagged peptides were fractionated by reversed-phase chromatography and were identified and quantified using a high-resolution Orbitrap mass spectrometer coupled with a Dionex Ultimate 3000 RSLC nano-UPLC (Thermo Fisher Scientific) for peptide separation. Proteomic data sets were analysed with the empirical Bayes moderated T-test implemented by the Limma package [30]. *P*-values were corrected for multiple hypothesis testing using the Benjamini–Hochberg method (FDR≤0.05). Differential expression was calculated on normalized log_2_ ratios of abundance. The MS/MS fragmentation data were searched against the National Centre for Biotechnology Information database using the MASCOT (Matrix Science) search engine.

### Metabolite analyses

The Tn::*argA* mutant (PW9762), AMB128 (the Tn::*argA ambA*^Ala128Val^) and MPAO1 (the progenitor strain of PW9762 and AMB128) were grown for 5 h in LB broth with good aeration. Aliquots (10 ml volume) were sedimented (4,000 ***g***, 20 °C, 5 min), and 5 ml samples of the clarified supernatant were filter-sterilized (0.22 µm filters) and frozen at −20 °C. The cell pellets were resuspended in 10 ml of 0.9% (wt/v) saline, then re-pelleted. The pelleted cells were then resuspended in 10 ml of a 2 : 2 : 1 mixture of methanol/acetonitrile/water, and the suspension was stored at −20 °C until the next day. The suspended/thawed cells were sonicated (3 mm tip sonicator, Branson, full power) for 60 s. The sonicated samples (*N*=4 for each strain) were then chilled to 0 °C and clarified by sedimentation (4,000 ***g***, 4 °C, 10 min). Aliquots (5 ml volume) of the clarified supernatant were removed and placed into new Universal tubes. The solvent was then gently evaporated at room temperature in an anaerobic chamber (to exclude oxygen during the drying-down process).

For metabolite analysis, the dried extracts were resuspended in 500 µl of cold (−20 °C) methanol/acetonitrile/water (2 : 2 : 1 by volume)+0.1% formic acid (FA) and dried. Before analysis, all samples received were reconstituted with 150 µl of H_2_O/acetonitrile 97.5 : 2.5+0.2% FA, vortexed and transferred to inserts. Pooled quality control of all samples was then generated by pooling 10 µl of the first replicate for each experimental condition and was injected every sixth sample. Targeted metabolomics analysis was performed using an Agilent 1290 LC system (Agilent Technologies, CA, USA) coupled to a QTRAP 4000 MS system (SCIEX, Danaher, WA, USA). Chromatographic separation was achieved using a Luna Omega Polar C18 column (Phenomenex, Danaher, WA, USA). The analysis was conducted in positive ionization mode (A: H_2_O+0.2% FA; B: acetonitrile+0.2% FA) on a 20 min gradient at 0.450 ml min^−1^ flow rate. All data were acquired in multiple reaction monitoring mode based on in-house assignments. Resulting spectra analysed using an in-house data analysis workflow based on [31].

## Results

### A PA mutant containing a Tn insertion in *argA* is auxotrophic for arginine

We screened a selection of arginine biosynthetic gene mutants from the UWGC PAO1 Tn library [19] to identify those that were unable to grow on minimal glucose medium in the absence of exogenous arginine. Where possible, care was taken to select mutants in which the Tn insertion was near to the 5′ end or middle of each ORF, to increase the probability of disrupting the function of the rest of the ORF. One such auxotroph (PW9762) contained a Tn insertion in *argA*. PW9762 contains an IS*phoA*/hah insertion (reverse orientation) at position 155/1299 (nt numbering) in *argA* (locus tag, PA5204). The IS*phoA*/hah insertion in *argA* was confirmed by PCR/Sanger sequencing (Fig. S1, available in the online Supplementary Material), and later by WGS (*see below*). Unlike the wild-type progenitor, PW9762 could not grow on MOPS-glucose minimal agar plates (Fig. 2a) in the absence of exogenous arginine. However, growth of the Tn::*argA* mutant in minimal medium could be restored following complementation with a copy of the wild-type *argA* gene supplied *in trans* on pUCP20 (Fig. 2b, c).

**Fig. 2.**
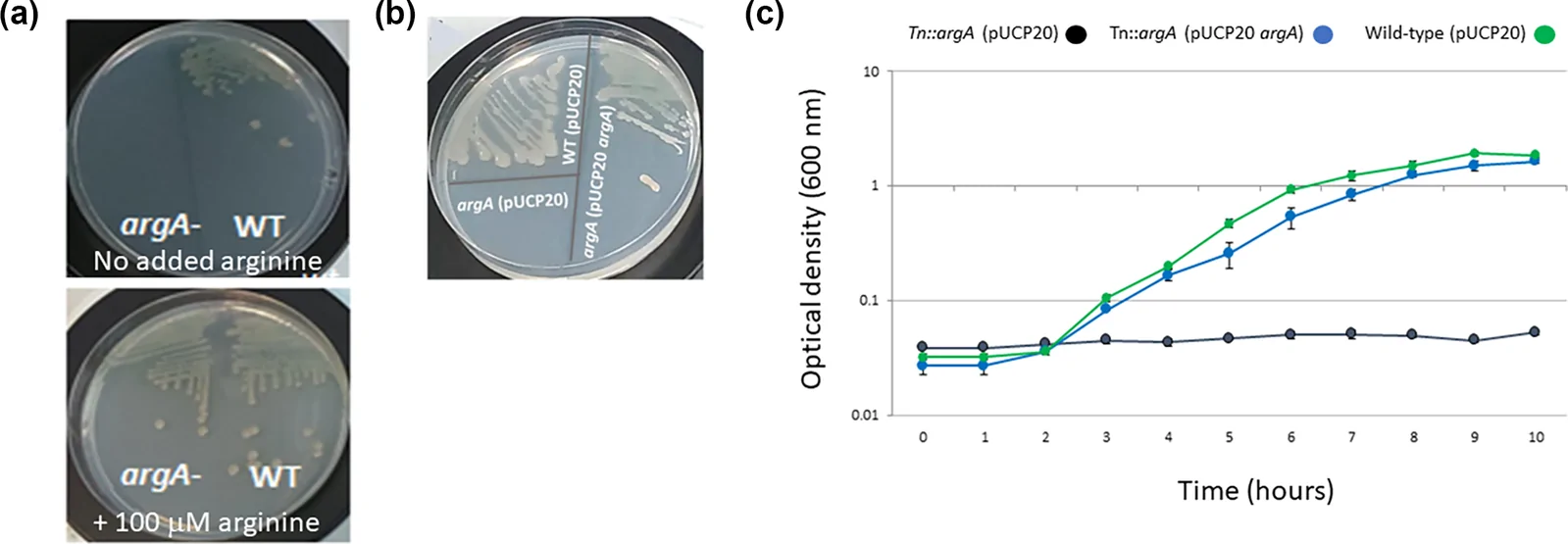
The Tn::*argA* mutant PW9762 is auxotrophic for arginine. (a) Chemical complementation of arginine auxotrophy in the Tn::*argA* mutant. Minimal medium agar (1.5% wt/v) plates buffered with MOPS were prepared ±100 µM L-arginine. The right-hand half of each plate was struck with wild-type PAO1, whereas the left-hand half was struck with the Tn::*argA* mutant (PW9762), as indicated. Note the absence of growth in the Tn::*argA* mutant in the absence of exogenous arginine. (b) Genetic complementation of the Tn::*argA* mutant on agar. Minimal MOPS-glucose medium plates (with no added arginine) were struck with PAO1 (pUCP20), Tn::*argA* (pUCP20) and Tn::*argA* (pUCP20*argA*). Note the restoration of prototrophy in the complemented strain. (c) Growth of the Tn::*argA* mutant in liquid culture. The figure shows growth curves of PAO1 (pUCP20), Tn::*argA* (pUCP20) and Tn::*argA* (pUCP20*argA*) in liquid MOPS-glucose minimal medium. Growth curves represent the mean±sd of three independent experiments.

### The Tn::*argA* auxotroph, PW9762, can re-acquire prototrophy through spontaneous ‘bypass’ mutations

Although cultures of PW9762 failed to grow overnight on agar plates containing minimal medium, after a few days on the bench, we noted that spontaneous prototrophs appeared with a frequency of ~7×10^−5^ prototrophs per c.f.u. originally plated (Fig. S2). These prototrophs manifested a variety of colony morphologies (Fig. S2B), perhaps suggesting involvement of different genes. Similarly, after prolonged (>24 h) incubation in liquid MOPS-glucose minimal medium, cultures of PW9762 manifested growth. By contrast, incubation of another arginine auxotroph (PW6991, containing a Tn insertion in the *argF* ORF) failed to give rise to growth in liquid culture, even after several days (Fig. S3A). To investigate the possible molecular-genetic basis of the mutations giving rise to arginine prototrophy in PW9762, we designed an experimental protocol (flow scheme in Fig. S3B) that would allow us to obtain pure isolates of these prototrophs for further study. WGS of one such arg^+^ isolate revealed that, in addition to the Tn insertion in *argA*, the genome contained just a single SNP (a C→T transition in *ambA*, leading to an Ala128→Val substitution in the encoded protein). This SNP was confirmed by PCR amplification of the *ambA* region and Sanger sequencing of the amplicon. (Some additional potential ‘SNPs’ were also identified in the WGS data, but subsequent PCR amplification of the regions around these ‘SNPs’ and Sanger sequencing of the PCR products revealed these to be false calls associated with poor-quality Illumina reads at the ends of contigs.) Hereafter, this Tn::*argA* bypass mutant is denoted AMB128. AmbA is a predicted membrane protein, and Deep Transmembrane Helices; Hidden Markov Model (TMHMM) analyses indicated that residue Ala128 is located in a region predicted to be exposed to the periplasm (Fig. 3a, b). The same mutation was also identified in one other independently isolated arg^+^ bypass mutant (‘DM2’). However, PCR amplification and Sanger sequencing of *ambA* from a selection of additional spontaneous *argA* bypass mutants failed to reveal further *ambA* mutants. We conclude that mutation(s) in *ambA* are just one of (presumably) many ways in which prototrophy can be restored to the *argA* mutant.

**Fig. 3.**
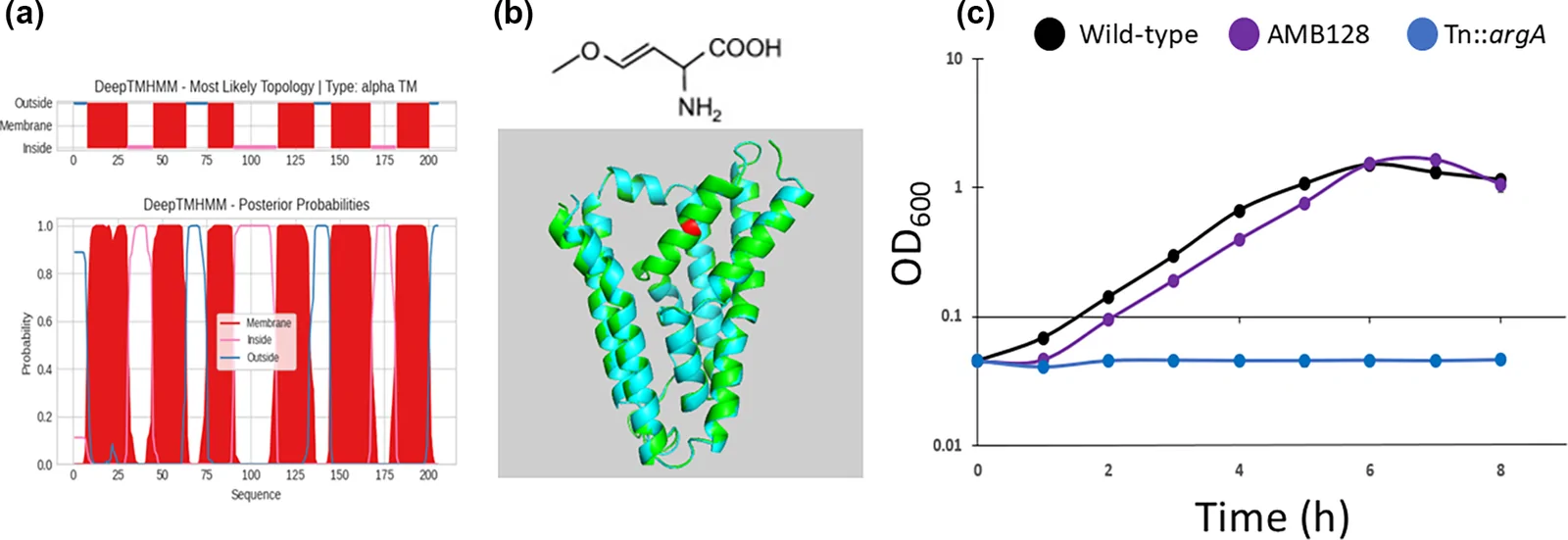
An SNP giving rise to the amino acid substitution Ala128→Val in AmbA bypasses the arginine auxotrophy in a Tn::*argA* mutant. (a) Deep TMHMM model of AmbA showing six predicted transmembrane helices. (b) Structure of l-2-amino-4-methoxy-*trans*-3-butenoic acid (AMB) and a three-dimensional structural model (AlphaFold3) of the AMB transporter, AmbA. The location of residue 128 is shown in red. The wild-type protein is coloured green, and the Ala128→Val variant cyan. The RMSD of the overlay (1,204/1,563 atoms across 205 amino acid residues) is 0.107 Å. (c) Growth of the wild-type (MPAO1), AMB128, and the Tn::*argA* mutant in MOPS-glucose minimal medium.

We also managed to revive two of the original *argA* auxotrophs characterized by Haas *et al.* [18] (Fig. S4). These mutants, PAO366 (carrying the *argA36* allele) and PAO540 (carrying the *argA171* allele, as well as *his-5075* and *cys-5605* alleles), manifest zero NAGS activity [18] and failed to yield spontaneous prototrophs, even after prolonged incubation on minimal medium agar plates. These data suggest that the appearance of spontaneous prototrophs in PW9762 (the Tn::*argA* mutant) is associated with this particular Tn insertion. Curious as to the exact nature of the mutations in *argA* in PAO366 and PAO540, we used PCR to amplify the *argA* ORF from each strain, and sequenced the products. Both strains contained mutations located in the catalytic ‘Gcn5-related N-acetyltransferase’ (GNAT) domain of the enzyme. The PAO366 strain (*argA36* allele) contained a T→C transition at position 1172 in the ORF, giving rise to a Thr→Ile substitution at position 391 in the encoded protein. The PAO540 strain (*argA171* allele) contained a deletion of the dinucleotide CG (the terminal CG in a series of five contiguous CG dinucleotides in the wild-type *argA* sequence) at position 1139 in the ORF. This led to a frameshift after Arg 380 and the consequent introduction of a premature stop codon (TAG) at position 404 of the encoded protein.

AmbA belongs to the LysE/LeuE family of transmembrane translocators and is predicted to export the anti-metabolite l-2-amino-4-methoxy-*trans*-3-butenoic acid (AMB) (Fig. 3b). Like other oxyvinylglycines, AMB irreversibly inhibits pyridoxal phosphate-dependent enzymes in bacteria and in higher organisms such as plants [32]. The AMB biosynthetic gene cluster *ambABCDE* was identified in PA through Tn mutagenesis and reverse genetics, and deletion of the *ambA* ORF leads to the abolition of AMB production [33]. Based on this, AmbA has been proposed to be involved in AMB export, although it forms a distinct predicted transcriptional unit compared with the rest of the *ambBCDE* cluster. AmbB and AmbE are non-ribosomal peptide synthetases, whereas AmbC and AmbD are predicted to be iron (II)/α-*keto*glutarate-dependent oxygenases [20]. There is no obvious reason why the Ala128→Val substitution in AmbA should restore arginine prototrophy to an *argA* mutant, although we note that in *Escherichia coli*, other LysE homologues include the ArgO/YggE arginine transporters involved in control of the arginine:lysine ratio in the cell [34].

### The *ambA^A128V^* allele is recessive

We first confirmed that the Tn::*argA ambA*^A128V^ mutant, AMB128, could grow in minimal medium in the absence of arginine without any appreciable lag phase. It did (Fig. 3c). Next, we examined whether the Ala128→Val mutation was dominant or recessive compared with the wild-type *ambA* allele (with the caveat that the term ‘wild-type’ is ambiguous, on many levels [35]). We did this by PCR-amplifying the *ambA* ORF (inclusive of 70 bp of DNA upstream from the start codon) from the PAO1 progenitor strain and from the AMB128 mutant, and cloning each amplicon (separately) into pUCP20. The pUCP20 containing *ambA*^WT^, *ambA*^Ala128Val^ or no insert, was then introduced into AMB128 and the ability of the resulting strain to grow in MOPS-glucose minimal medium was measured (Fig. 4). The AMB128 containing pUCP20 alone, or pUCP20(*ambA*^Ala128Val^) grew robustly. However, the AMB128 containing pUCP20(*ambA*^WT^) did not. We conclude that the wild-type *ambA* allele is dominant compared with the *ambA*^Ala128Val^ allele. To further test this hypothesis, we introduced (separately) the mutated and wild-type *ambA* allele (on pUCP20) into the original *argA* mutant. The *argA* mutant should still contain a wild-type (i.e. dominant) copy of the *ambA* gene, so based on the data in Fig. 4, we did not expect to see restoration of prototrophy. Indeed, we did not (Fig. S5). Given that the *ambA*^Ala128Val^ mutation appears to elicit CoF (or possibly, an EoF) of the gene rather than a loss of function (see below), the dominance of the wild-type allele is both noteworthy and unexpected.

**Fig. 4.**
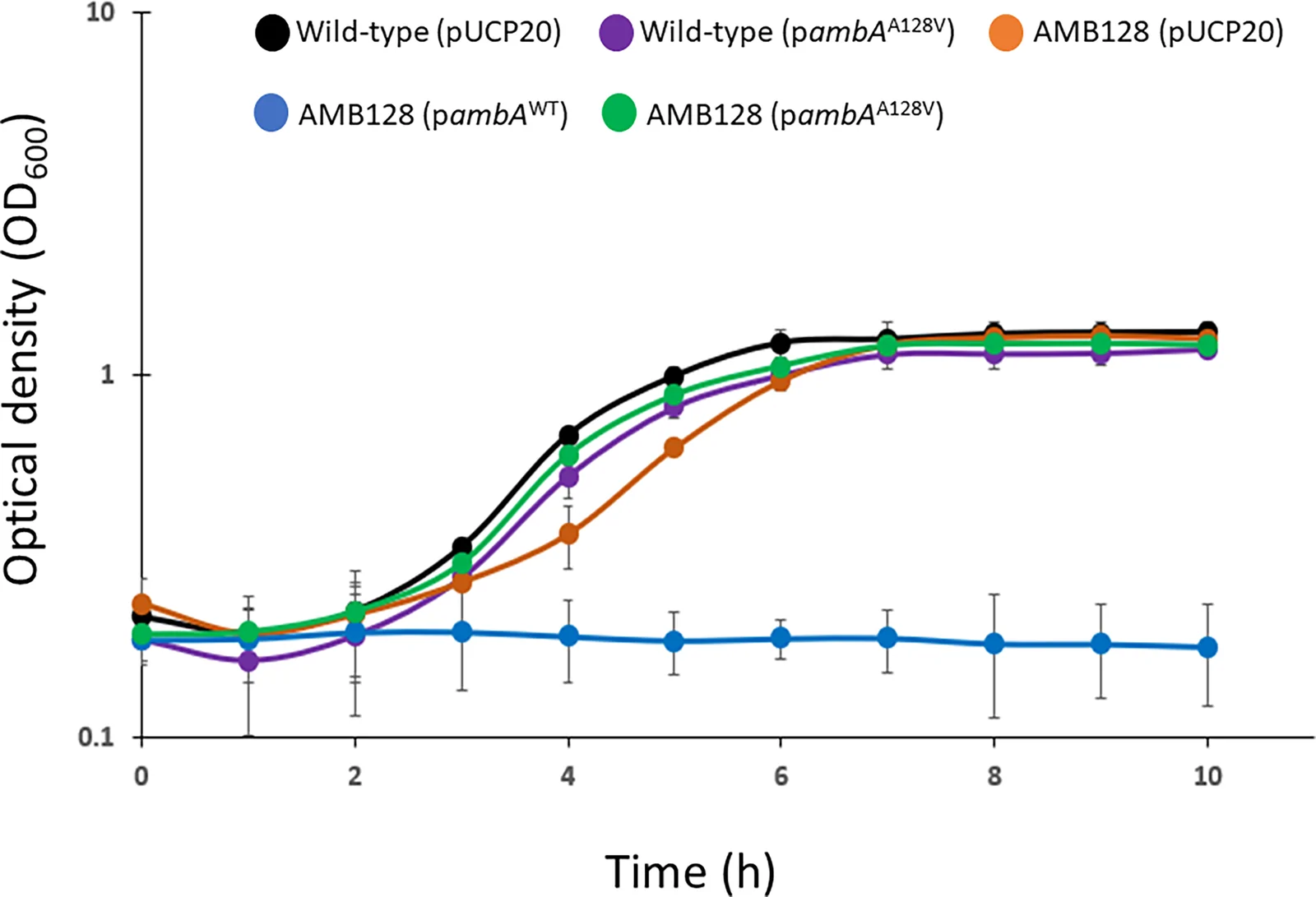
Complementation of AMB128 with plasmid-borne *ambA* alleles reveals that the wild-type *ambA* allele is dominant. The *ambA* ORF was PCR-amplified from PAO1 or from AMB128, and the amplicons were cloned into pUCP20. The resulting p*ambA*^WT^ and p*ambA*^Ala128Val^ plasmids (or empty pUCP20) were introduced into wild-type MPAO1 or AMB128, as indicated, and growth was assessed on MOPS-glucose minimal medium.

### The *ambA^A128V^* allele does not lead to loss of function

*In silico* prediction using PROVEAN [36] suggested that the *ambA*^Ala128Val^ mutation may be deleterious and could lead to loss of gene function. To investigate this further, we generated a Tn::*argA* Δ*ambA* double mutant. This was done by using a transducing phage, ΦPA3 [37], to transfer the Tet^R^ marker (associated with the Tn5 insertion) from the UWGC-derived Tn::*argA* mutant (PW9762) into a Δ*ambA* recipient [38]. One of the transductants was selected and PCR/sequencing was used to confirm that it contained both the in-frame deletion in *ambA* and the Tn insertion in *argA*. It did (Fig. S1). However, and as with the original Tn::*argA* mutant, the Tn::*argA* Δ*ambA* double mutant failed to grow in MOPS-glucose minimal medium (Fig. 5a). This indicates that loss of *ambA* function does not confer prototrophy to the Tn::*argA* mutant. By contrast, the Δ*ambA* mutant, AMB128 (the Tn::*argA ambA*^Ala128Val^ double mutant), and the wild-type strains MPAO1 (the immediate ancestor of the UWGC-derived Tn::*argA* mutant) and LPAO1 (the immediate ancestor of the Δ*ambA* mutant [38]) all grew in this medium (Fig. 5a). These data indicate that the *ambA*^Ala128Val^ mutation does not lead to loss of gene function.

**Fig. 5.**
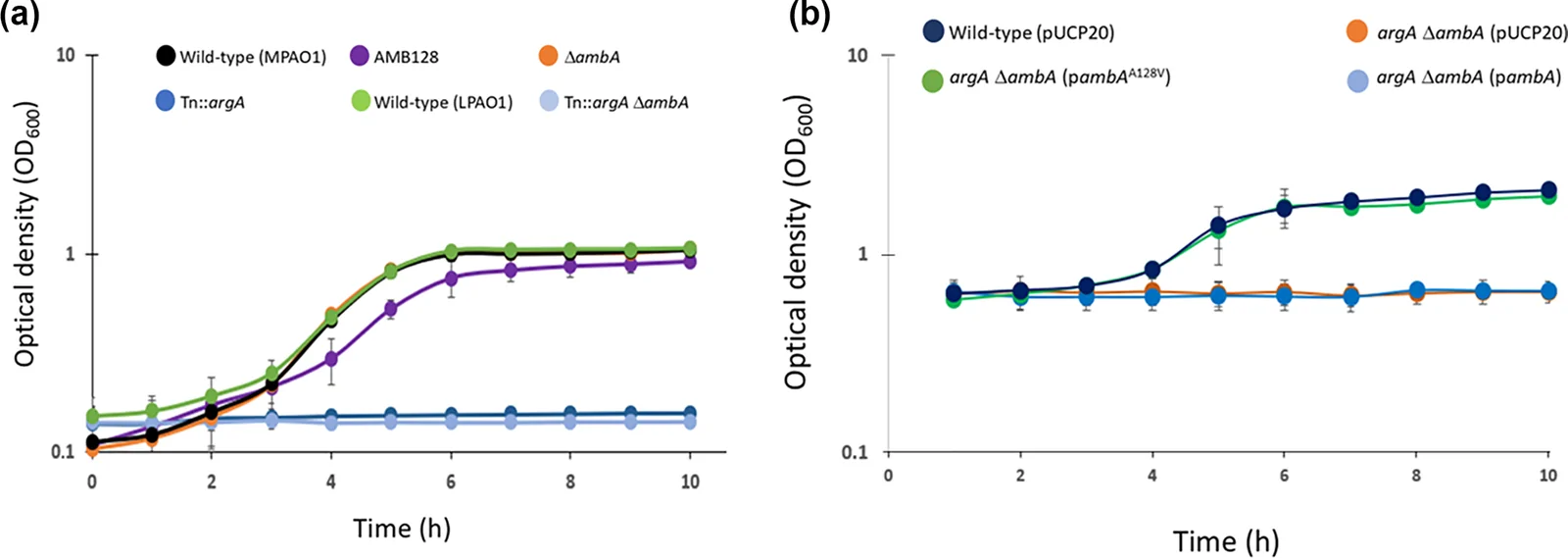
(a) The *ambA*^Ala128Val^ mutation restores prototrophy to the Tn::*argA* mutant, whereas the Δ*ambA* mutation does not. Growth (monitored as OD_600_ of the indicated cultures) was measured in MOPS-glucose minimal medium. The wild-type progenitors of the Tn::*argA* auxotroph and of the Δ*ambA* mutant are MPAO1 (Manoil PAO1) and LPAO1 (Li lab PAO1), respectively. OD values are expressed as mean±sd, based on *N*=3 independent biological replicates. (b) Growth of the Tn::*argA* Δ*ambA* double mutant complemented with plasmid-borne *ambA* from the wild-type progenitor or from the AMB128 mutant. The cultures were grown in MOPS-glucose minimal medium with good aeration at 37 °C. The OD_600_ of the cultures was measured hourly, and the results are expressed as the mean±sd for *N*=3 independent biological replicates. The plasmids pUCP20, p*ambA* and p*ambA*^A128V^ represent the empty pUCP20 vector, recombinant pUCP20 vector carrying *ambA* cloned from PAO1 and recombinant pUCP20 vector with *ambA* cloned from AMB128, respectively. Note that growth is arrested in the Tn::*argA* Δ*ambA* double mutant when complemented with empty pUCP20 or p*ambA*. By contrast, prototrophy is restored when the double mutant is complemented with mutated *ambA*^A128V^.

We next examined the effect of re-introducing either wild-type *ambA* or *ambA*^Ala128Val^ on a plasmid into the Tn::*argA* Δ*ambA* mutant (Fig. 5b). The Tn::*argA* Δ*ambA* mutant carrying pUCP20 alone or pUCP20(*ambA*) retained arginine auxotrophy. However, introduction of pUCP20-borne *ambA*^Ala128Val^ restored arginine prototrophy. We conclude that the Ala128→Val substitution in AmbA gives rise to a CoF or EoF of the encoded protein.

### The AMB128 (Tn::*argA ambA*^Ala128Val^) proteome manifests changes associated with dysregulation of arginine or a related metabolite

To gain further insight into how the CoF associated with the *ambA*^Ala128Val^ mutation might contribute towards bypassing arginine auxotrophy in the Tn::*argA* mutant, we used tandem mass tagging (TMT) and liquid chromatography (LC)–MS/MS to compare the proteome of MPAO1 (the progenitor strain of the Tn::*argA* mutant, PW9762) with that of the Tn::*argA ambA*^Ala128Val^ mutant, AMB128. Protein was extracted from cultures grown in MOPS-glucose minimal medium and harvested in the exponential phase of growth. We were able to identify peptides from 3,758 ORFs. The biological replicates of the strains clearly segregated in principal component(s) analysis (PCA) (Fig. S6A). For differential expression analyses, proteins were considered significantly modulated if they displayed ≥2-fold modulation with a False Discovery Rate (FDR)-adjusted *P*-value of ≤0.05 (Fig. 6). A list of the top 20 most significantly up- and down-regulated proteins in AMB128 (cf. MPAO1) is given in Tables S1 and S2, respectively. Out of interest, we also quantified the proteome of the Δ*ambA* mutant (cf. its progenitor, LPAO1) but this comparison revealed rather few and modest modulations (Fig. S6B). This indicates that (i) in these growth conditions, loss of *ambA* function has very little impact on cell physiology and (ii) that the majority of the proteomic changes associated with AMB128 arise either as a consequence of the original *argA* mutation or from the *ambA*^Ala128Val^ mutation. Unfortunately, we could not deconvolute the latter possibility further because the arginine auxotrophy of the original Tn::*argA* mutant prevented it from growing in MOPS-glucose minimal medium.

**Fig. 6.**
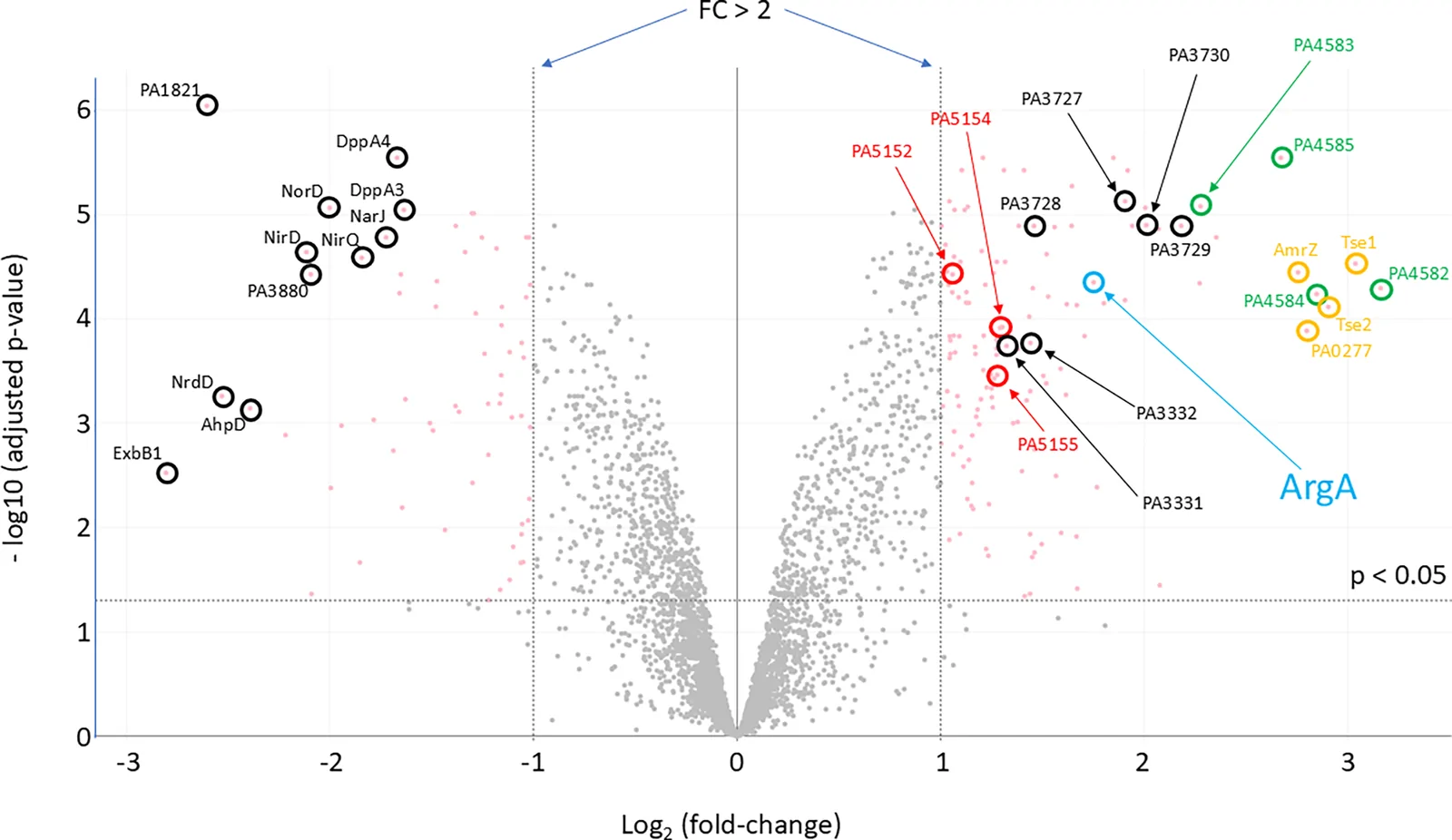
Volcano plot representing the log_2_ fold-change in protein abundance versus statistical significance for the cell-associated proteome of AMB128 and MPAO1. For ease of navigation, dotted lines delineating fold-change values of ±2 and a significance threshold of ≤0.05 are shown. Proteins on the left-hand side of the plot are down-modulated in AMB128, whereas proteins on the right-hand side are up-modulated in AMB128. A selection of the more highly modulated proteins on each side of the plot have been annotated. On the right-hand side, ORFs that are derived from the same operon are highlighted in the same colour, as are ORFs that are known to encode virulence-associated proteins (in yellow text). Samples were harvested for proteomics from *N*=3 independent biological cultures of each strain.

Among the most highly up-regulated proteins in AMB128 were gene products from the PA4582–PA4585 cluster, encoding the Rtc ‘healing and sealing’ RNA repair functions. Up-regulation of these proteins is suggestive of RNA-associated stress. In this regard, a gene annotated as tRNA^arg4^ (PA4581.1) is located in the intergenic region between *rtcR* (PA4581) and the divergently transcribed *rtc* cluster. tRNA^arg4^ has a CGU anti-codon and retains the ‘arginine discriminator’ base (G_73_) towards the 3′ end of the molecule [39]. However, unlike the other five tRNA^arg^ variants encoded by PA, tRNA^arg4^ lacks a -CCA charging motif at the 3′ end of the molecule and has a guanine instead of the usual adenine associated with arginyl-tRNAs at position 20 in the D loop. These unusual features, and the obvious potential link between tRNA^arg4^ and arginine metabolism, made us wonder whether tRNA^arg4^ expression might be linked with expression of the *rtc* genes in AMB128. To investigate this further, we usedquantitative-reverse transcription polymerase chain reaction to monitor the levels of tRNA^arg4^ [and, as controls, levels of a ‘canonical’ tRNA^arg^, tRNA^arg2^ (PA0263.1) and of a tRNA that is presumably unrelated to arginine metabolism, tRNA^ala3^ (PA4280.3)] in MPAO1 and AMB128 (Fig. S7). Although tRNA^arg4^ levels were lower (by ~50%) than those of tRNA^arg2^ and tRNA^ala3^, they were similar in both genetic backgrounds. We conclude that, in spite of its location within the *rtc* cluster, aberrant expression of tRNA^arg4^ per se is unlikely to be responsible for induction of the *rtc* genes.

Additional up-regulated proteins included PA3728–PA3732 (encoding a cluster of ORFs of unknown function), several proteins involved in Type VI Secretion (T6S), and a cluster of ORFs (PA5152–PA5155) involved in arginine/lysine/histidine/ornithine/octopine transport. The sequence immediately upstream of PA5152 is predicted to contain an ArgR binding site, and indeed, the expression of this gene cluster has been previously shown to be stimulated by ArgR in the presence of arginine [40]. ArgR is an AraC-type regulator that represses arginine biosynthesis and induces arginine catabolism (fermentation), especially when oxygen is limiting [40]. The increased abundance of proteins encoded by PA1552–PA1555 in AMB128 is therefore suggestive of elevated arginine (or a closely related metabolite). In this regard, arginine catabolism is known to be inversely regulated with denitrification [41]. Consistent with this, among the down-regulated proteins, we noted several involved in denitrification, including proteins from the Nir, Nar and Nor sub-systems, as well as dipeptide-binding proteins (DppA3 and DppA4) and a protein involved in the response to nitrosative stress (Fhp) (Fig. 6). Finally, it is worth noting that a γ-glutamylpolyamine synthetase, PauA4, is among top 20 up-regulated proteins in AMB128. Polyamines accumulate when intracellular arginine levels are high and lead to growth arrest unless they can be removed. PauA4 assists in this detoxification by γ-glutamylating putrescine (a polyamine) in the first step of a pathway that ultimately yields γ-aminobutyrate (GABA).

Unexpectedly, the proteomic analyses also identified peptides corresponding to ArgA in extracts of AMB128. However, the ArgA-derived peptides from AMB128 all mapped downstream of the tryptic cleavage site (Arg189) in the middle of the protein, rather than to the upstream part of the ORF (Fig. S8). (Trypsin was used to generate the peptides in the proteomics analysis pipeline.) Interestingly, even in the MPAO1 samples, we mapped only one peptide upstream of Arg189, indicating that the N-terminal portion of full-length ArgA may be intrinsically unstable and readily degraded by proteases. We also note that the ArgA-derived peptides in AMB128 were ~two- to threefold more abundant than those in MPAO1. These observations raised the possibility that AMB128, and its precursor, the Tn::*argA* mutant, might retain some NAGS activity. To test this, we assayed NAGS activity in cell extracts of LB-grown MPAO1, the Tn::*argA* mutant and AMB128. All three strains manifested robust NAGS activity (Fig. 7a).

**Fig. 7.**
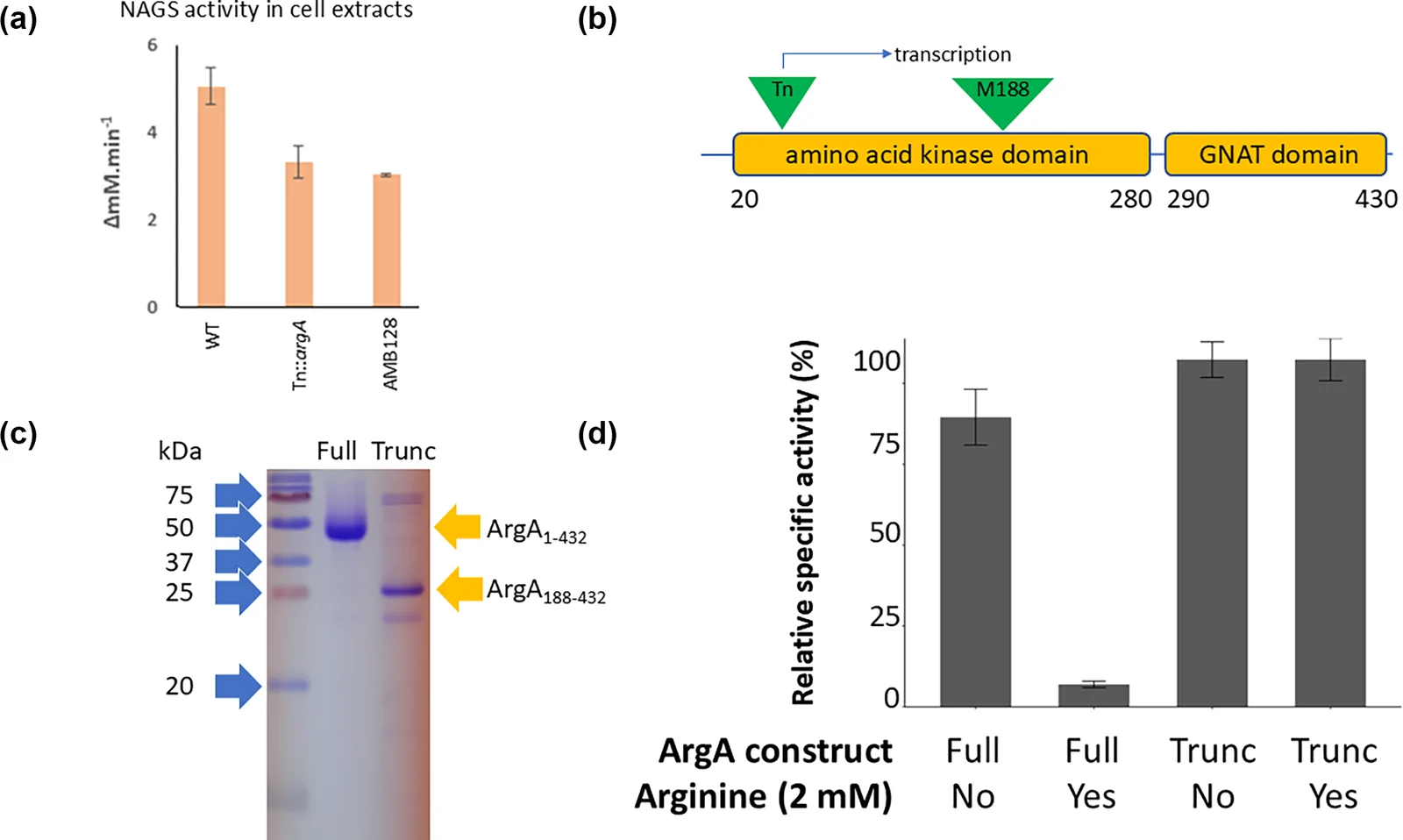
The Tn::*argA* and AMB128 mutants retain NAGS activity. (a) NAGS activity in cell extracts. Cultures of the indicated strains were grown until stationary phase with good aeration at 37 °C in LB. The cells were then pelleted, washed (in PBS) and lysed by sonication. The lysates were clarified by sedimentation (14,500 ***g***, 5 min, 4 °C), and their protein content was determined using Bradford assay. After normalizing the protein content in each extract, NAGS activity was measured in the presence of saturating l-glutamate (15 mM) and acetyl-CoA (3 mM). Data represent the mean±sd of *N*=3 biological replicates. (b) The Tn insertion in PW9762 (Tn::*argA*) and AMB128 disrupts the amino acid kinase (AAK) domain of ArgA. An outward-facing neomycin phosphotransferase promoter in the inserted IS*phoA*/hah means that the downstream the GNAT domain (responsible for NAGS activity) is potentially translated from an alternative start codon (Met188 in the full-length protein). (c) Purification of N-terminally His_8_-tagged full-length ArgA (ArgA_1-432_) and truncated ArgA (ArgA_188-432_). Both proteins were purified by Ni-NTA chromatography from the soluble fraction of *E. coli* cell extracts over-expressing the relevant transgene. Note that although the full-length protein could be purified to near homogeneity, the truncate contained some contaminants, even after extensive washing in high-salt buffer. (d) NAGS activity in the Arg_188-432_ mutant is not feedback inhibited by arginine. The NAGS activity associated with the purified full-length (ArgA_1-432_) and truncated (ArgA_188-432_) ArgA was measured in the presence and absence of 2 mM l-arginine. Data represent the mean±sd of *N*=3 independent technical replicates. Specific activity was normalized per mol of each enzyme present, and values are reported relative to the highest measured specific activity.

### The Tn insertion in Tn::*argA* and AMB128 generates a truncated ArgA protein that retains NAGS activity but is insensitive to inhibition by arginine

Given the abundance of C-terminal ArgA-derived peptides in AMB128 and the measurable NAGS activity in cell extracts of the Tn::*argA* and AMB128 strains, this prompted us to examine in more detail the possible consequences of the Tn insertion on ArgA function. The Tn was inserted in *argA* at position 52/433 in the amino acid sequence, thereby disrupting the amino acid kinase (AAK) domain (residues 25–280) (Fig. 7b). The AAK domain is regulatory and binds the pathway end-product, arginine, which strongly inhibits the full-length enzyme [42, 43]. C-terminal to the AAK domain is the GNAT domain (residues ~290–430) responsible for catalysing the acetylation of glutamate. However, the Tn IS*phoA*/hah sequence that disrupts the AAK domain contains an outward-facing neomycin phosphotransferase promoter, which can potentially drive expression of the downstream DNA, including the GNAT domain. This promoter was deliberately engineered into the Tn to reduce potential polar effects of the Tn insertions [19]. Closer inspection of this downstream DNA revealed several potential start codons, one of which (Met188) was preceded by a potential ribosome-binding site (AAGACGX_5_ATG) (Fig. S8). For comparison, the ribosome-binding site of the full-length *argA* ORF is AGGCTTX_8_ATG. This raised the possibility that AMB128 and the Tn::*argA* mutant might express a truncated form of NAGS (ArgA_188-432_) that is insensitive to feedback inhibition by arginine. To test this, we over-expressed (in *E. coli*) and purified full-length, N-terminally His_8_-tagged PA ArgA (ArgA_1-432_) and N-terminally His_8_-tagged truncated PA ArgA_188-432_ (Fig. 7c). The purified enzymes were then assayed for NAGS activity in the presence and absence of 2 mM arginine. Whereas arginine strongly inhibited full-length ArgA (as previously reported [42, 43]), it had little effect on the truncated ArgA (Fig. 7d). We conclude that the Tn insertion in PW9762 and in AMB128 likely leads to elevated expression of a constitutively active ArgA_188-432_ protein. For reasons that are not clear, this dysregulated (at both genetic and enzymatic levels) ArgA leads to arginine auxotrophy.

### The Tn::*argA* and AMB128 mutants have an altered metabolome

Since the proteomic analyses indicated that AMB128 (and by extrapolation, possibly also the Tn::*argA* mutant, PW9762) may contain elevated levels of intracellular arginine, we next examined the metabolic profile of AMB128 and the Tn::*argA* mutant (cf. the progenitor strain, MPAO1). This was done by growing each strain to mid-exponential phase in LB and then preparing methanol/acetonitrile/water extracts of the lysed cells. Metabolites were assessed in each extract using LC–MS. PCAs of the metabolite profiles in the cell extracts showed clear separation of the MPAO1 samples from the Tn::*argA* and AMB128 samples (Fig. 8a). Closer inspection of the data revealed that trimethylated compounds such as N6-trimethyllysine, betaine and (phosphoryl-)choline were more abundant in the Tn::*argA* and AMB128 cell extracts compared with the MPAO1 extracts, as were levels of histidine and histamine (Fig. 8a, b and Datasets S1 and S2). Presumably, these changes are linked, either directly or indirectly, with the constitutively active NAGS activity in these cell extracts.

**Fig. 8.**
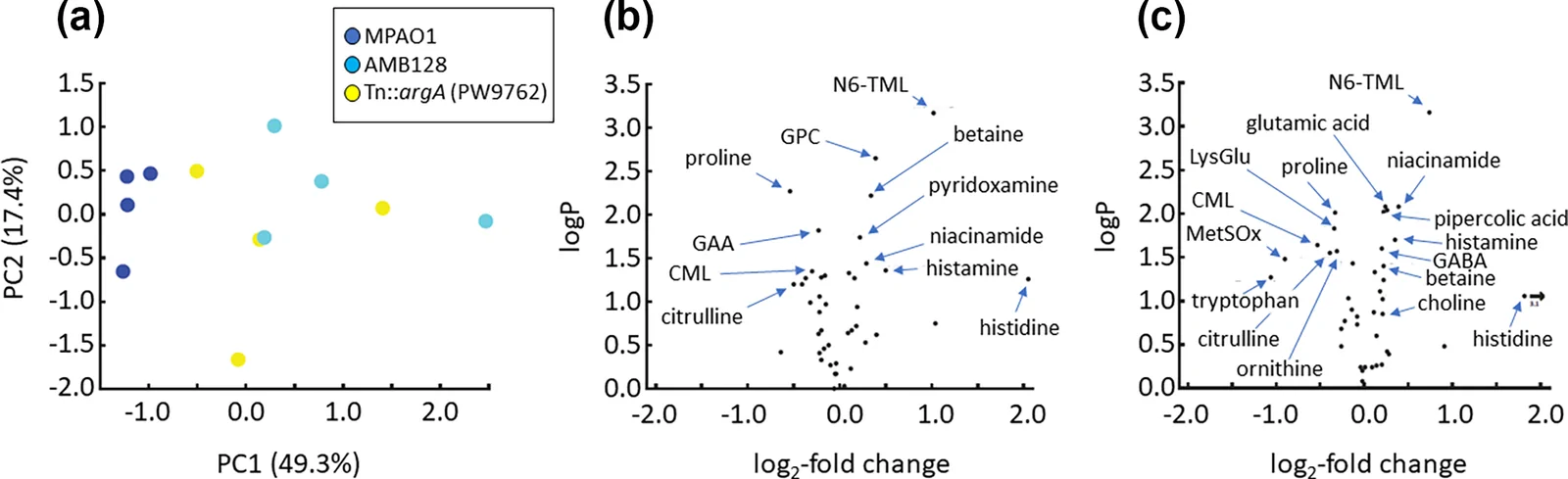
The *ambA*^A128V^ mutation does not restore the metabolome of AMB128 to a wild-type state. (a) PCA of metabolites quantified from the cell extracts of MPAO1 (●), PW9762 (Tn::*argA*) (●) and AMB128 (●). (b) Volcano plot showing metabolite levels in MPAO1 compared with PW9762 (Tn::*argA*). Metabolites on the right-hand side of the plot (positive log_2_-FC) had higher levels in the Tn::*argA* mutant, whereas metabolites on the left-hand side of the plot (negative log_2_-FC) had higher levels in MPAO1. (c) Volcano plot showing metabolite levels in MPAO1 compared with AMB128. Metabolites on the right-hand side of the plot (positive log_2_-FC) had higher levels in AMB128, whereas metabolites on the left-hand side of the plot (negative log_2_-FC) had higher levels in MPAO1. In both (b) and (c), key significantly modulated metabolites are labelled. Samples were harvested for metabolomics from *N*=4 independent biological cultures of each strain.

Given its restored arg^+^ phenotype, the co-clustering of the AMB128 samples with those of the Tn::*argA* mutant was somewhat surprising. Detailed inspection of the data revealed that the intracellular metabolite profile of the Tn::*argA* mutant was overall remarkably similar to that of AMB128, although we did note a significant elevation of glutamate and GABA in the AMB128. The increased levels of GABA in AMB128 extracts are consistent with the increased abundance of PauA4 (which γ-glutamylates the polyamine putrescine in the first step of its conversion to GABA) seen in the proteomic analyses.

We also analysed the metabolic footprint of each strain in its respective spent culture medium (and cf. the same medium before bacterial growth). These data indicated that the Tn::*argA* and AMB128 strains exhibited increased (relative to MPAO1) uptake of arginine from the growth medium. This increased uptake may contribute towards the high intracellular arginine levels inferred in AMB128 from the proteomic data. However, we note that the same strains also manifested increased uptake of threonine, serine, isoleucine and glutamic acid too (Dataset S2), indicating that the metabolic impact(s) of the Tn::*argA* insertion and *ambA*^A128V^ mutation are complex. Rather few changes clearly differentiated the Tn::*argA* mutant footprint from that of AMB128, although phosphorylcholine (but not choline) levels were much higher in the spent medium of the AMB128 culture compared with either MPAO1 or the Tn::*argA* mutant.

## Discussion

In this work, we investigate how the arginine auxotrophy associated with a dysfunctional ArgA protein can be overcome through acquisition of a secondary mutation in a gene not previously linked with arginine metabolism.

In spite of the fact that PW9762 is an arginine auxotroph and contains a Tn insertion near to the 5′ end of *argA*, an outward-facing promoter in the Tn leads to expression of an arginine-insensitive truncated ArgA enzyme (ArgA_188-432_) that retains robust NAGS activity. This raises the question of why this catalytically active mutant should be auxotrophic for arginine in the first instance. We also identify an SNP in a gene (*ambA*; annotated as a secondary metabolite transporter) that enables the arginine auxotrophy of the Tn::*argA* mutant to be bypassed. This raises the equally intriguing question of why a mutation in an ORF apparently unrelated to arginine metabolism can restore prototrophy. On a more general level, these data highlight two cautionary issues that are often overlooked. The first is that Tn insertions, even near to the start of an ORF, may not necessarily lead to complete loss of gene function, even though they may somewhat misleadingly manifest ‘the expected phenotype’ (in our case, arginine auxotrophy). Second, extragenic ‘bypass mechanisms’ that restore closer-to-wild-type function can be readily acquired.

Although the current study reveals why the specific Tn insertion in PW9762 has the *potential* for restoration of arginine prototrophy (re: the presence of a catalytically active ArgA truncate with NAGS catalytic activity, ArgA_188-432_), it is still not clear why this prototrophy is only realized in certain genetic backgrounds, such as AMB128. One explanation for the arginine auxotrophy of the PW9762 mutant is that excessive arginine synthesis arising from (i) the increased expression of ArgA_188-432_ driven by the Tn insertion and (ii) the dysregulation of ArgA_188-432_ leads to the buildup of a toxic pathway intermediate, pathway product or downstream metabolite. Presumably, the *ambA*^Ala128Val^ mutation somehow relieves this intoxication. A possible clue to the nature of this putative inhibitory compound(s) came from the proteomic analysis, which revealed elevated expression of a polyamine detoxification enzyme (PauA4) in AMB128. Polyamines such as putrescine, spermidine and spermine are essential for the optimal proliferation of many bacteria, but an excess of these compounds can inhibit protein synthesis and arrest cell growth (see [44] and references therein). Polyamines are generated from arginine, and excess arginine synthesis is directly linked with polyamine production. Therefore, one possibility is that, as a result of unrestrained flux through the arginine biosynthetic pathway in the Tn::*argA* mutant, polyamine poisoning prevents the growth of PW9762. However, this does not explain why the strain is dependent upon the presence of exogenous arginine for growth; in this regard, the mutant behaves like a ‘classical’ arginine auxotroph in which flux through the arginine biosynthetic pathway is limited. Clearly, and in spite of the apparently unrestrained NAGS activity of ArgA_188-432_, the situation in PW9762 is more nuanced. In this regard, when the ArgA-E pathway was first being characterized in the 1970s, Haas and Leisinger noted that NAGS activity is inhibited by polyamines [45], although detailed kinetic analyses to inform on the mechanism of this inhibition were not carried out. With purified ArgA in hand, we confirmed that spermine – and to a lesser extent, also spermidine – do indeed inhibit ArgA, but that unlike the inhibition by arginine (which is uncompetitive), inhibition by polyamines is competitive with respect to the substrate glutamate (Fig. S9). An economical hypothesis is that the arginine:polyamine level in the cell is normally held in check by the strong allosteric inhibition of NAGS by the pathway end-product, arginine (Fig. 9a). However, the loss of this allosteric inhibition as a result of the Tn insertion in PW9762, coupled with the greater expression level of the unregulated NAGS in this mutant, disrupts the intracellular arginine:polyamine ratio. High concentrations of polyamines in the cytoplasm of the Tn::*argA* mutant competitively block glutamate binding to NAGS, thereby lowering flux through the pathway to a steady-state level below the threshold required for prototrophy (Fig. 9b). The greater arginine content of AMB128 cells inferred from the proteomic data suggests that this inhibition of flux is relieved by the *ambA*^Ala128Val^ mutation in AMB128, possibly due to increased efflux or degradation of one or more polyamines, thereby restoring a more equitable arginine:polyamine ratio (Fig. 9c).

**Fig. 9.**
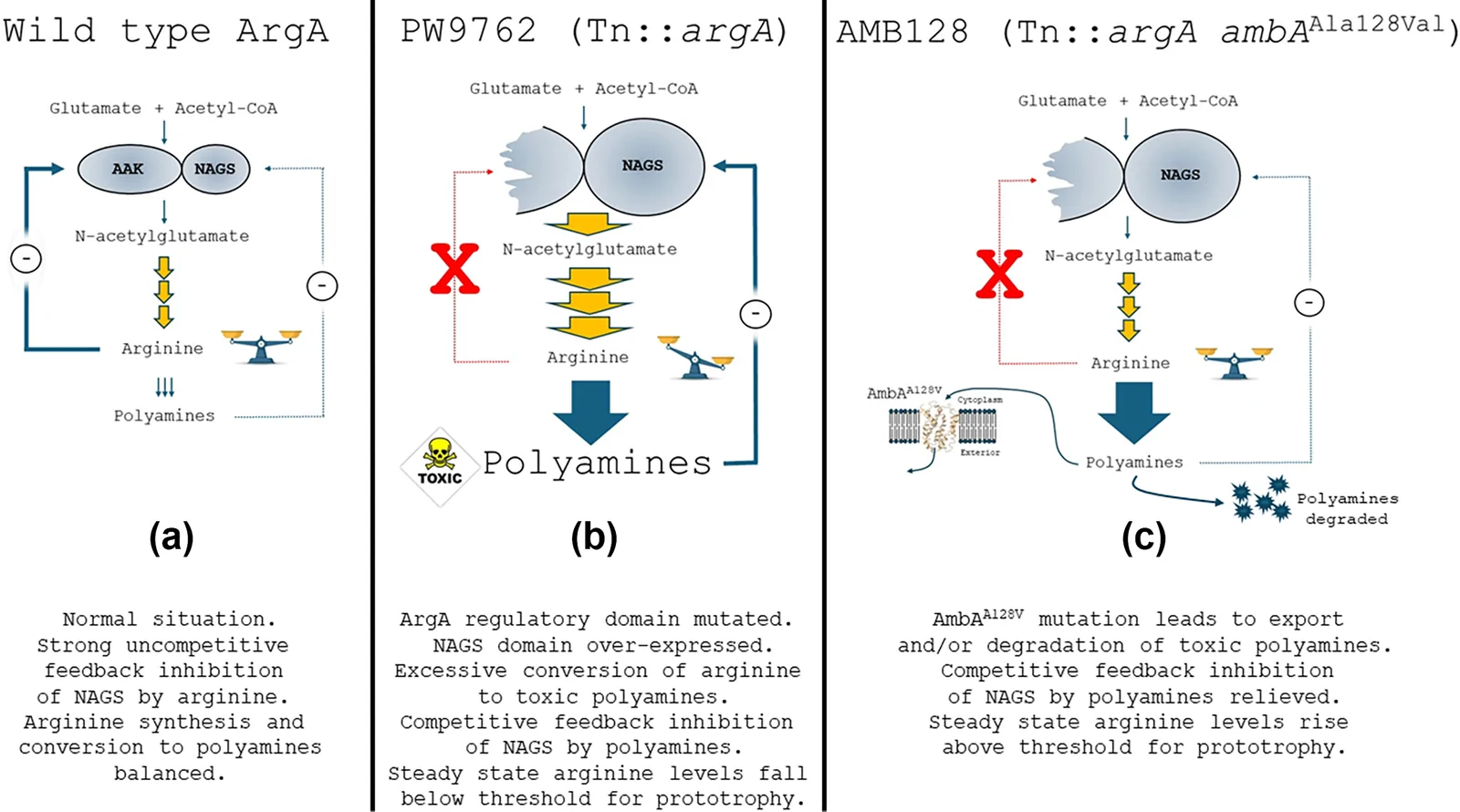
Model showing how the dysfunctional Tn::*argA* mutant may lead to elevated polyamine production, and how the *ambA*^A128V^ mutation may overcome polyamine toxicity. See text for details.

The model above offers a reasonable explanation for how the *ambA*^Ala128Val^ mutation might bypass the block on arginine synthesis in PW9762; by increasing polyamine breakdown or efflux. This is evidenced by the increased abundance of PauA4 and by the increased production (cf. the Tn::*argA* mutant) of GABA – a polyamine breakdown product – in the AMB128 mutant. The mechanistic basis for how the AmbA^Ala128Val^ substitution stimulates polyamine catabolism/removal is not clear. One obvious (but unproven) possibility is that, as an ArgO-family transporter, the Ala128→Val substitution expands the AmbA substrate specificity so that it exports, e.g. one or more polyamines, polyamine derivatives or polyamine precursors. In this regard, the dominance exhibited by the wild-type *ambA* allele is intriguing. One explanation for this may be that AmbA needs to be in a homomultimeric state for activity. In this scenario, ‘contamination’ of the mutant multimer by wild-type protomers may disrupt function. This mechanism implies highly concerted activity between the protomers of the putative multimer; an issue that remains to be tested.

On a more general point, our data reinforce the notion that although most SNPs are assumed to be either neutral or disruptive with respect to gene function (and indeed, probably are), they can also elicit CoF or EoF. Given the sheer amount of sequence space available to PA (or, for that matter, most other bacteria), we speculate that the matrix of evolutionary variation due to CoF/EoF mutations is likely to be under-appreciated. Indeed, of the many spontaneous Tn::*argA* prototrophs that we observed, just two verifiably contained a mutation in *ambA*, suggesting that multiple evolutionary ‘solutions’ are accessible to the organism to bypass this particular genetic block. Although WGS is invaluable in identifying such mutations, evidence for their CoF/EoF status still requires labour-intensive biochemical and genetic analyses; CoF/EoF mutations are currently impossible to predict computationally.

## Supplementary material

10.1099/mic.0.001760 Supplementary Material 1.

10.1099/mic.0.001760 Supplementary Material 2.

## Abbreviations

AAK: amino acid kinase
AMB: l-2-amino-4-methoxy-*trans*-3-butenoic acid
CF: cystic fibrosis
CoF: change-of-function
DTNB: 5′,5-dithiobis(2-nitrobenzoic acid)
EoF: expansion-of-function
FA: formic acid
FDR: False Discovery Rate
GABA: γ-aminobutyrate
gDNA: genomic DNA
GNAT: Gcn5-related N-acetyltransferase
LC: liquid chromatography
NAG: *N*-acetyl l-glutamate
NAGS: *N*-acetylglutamate synthase
Ni-NTA: Nickel-Nitrilotriacetic Acid
OAT: ornithine acetyltransferase
PA: *Pseudomonas aeruginosa*
PCA: principal component(s) analysis
TMHMM: Transmembrane helices; Hidden Markov Model
TMT: tandem mass tagging
Tn: transposon
WGS: whole-genome sequencing

## References

[1] Crone S, Vives-Flórez M, Kvich L, Saunders AM, Malone M (2020). The environmental occurrence of *Pseudomonas aeruginosa*. APMIS.

[2] Swain J, Askenasy I, Rudland Nazeer R, Ho PM, Labrini E (2025). Pathogenicity and virulence of *Pseudomonas aeruginosa*: recent advances and under-investigated topics. Virulence.

[3] Stapleton PJ, Izydorcyzk C, Clark S, Blanchard A, Wang PW (2021). *Pseudomonas aeruginosa* strain-sharing in early infection among children with cystic fibrosis. Clin Infect Dis.

[4] Moore MP, Lamont IL, Williams D, Paterson S, Kukavica-Ibrulj I (2021). Transmission, adaptation and geographical spread of the *Pseudomonas aeruginosa* Liverpool epidemic strain. Microb Genom.

[5] Iwańska A, Trafny EA, Czopowicz M, Augustynowicz-Kopeć E (2023). Phenotypic and genotypic characteristics of *Pseudomonas aeruginosa* isolated from cystic fibrosis patients with chronic infections. Sci Rep.

[6] Clark S, Diaz Caballero J, Cheang M (2015). Phenotypic diversity within a *Pseudomonas aeruginosa* population infecting an adult with cystic fibrosis. Sci Rep.

[7] Winstanley C, O’Brien S, Brockhurst MA (2016). *Pseudomonas aeruginosa* evolutionary adaptation and diversification in cystic fibrosis chronic lung infections. Trends Microbiol.

[8] Weimann A, Dinan AM, Ruis C, Bernut A, Pont S (2024). Evolution and host-specific adaptation of *Pseudomonas aeruginosa*. Science.

[9] Yang L, Jelsbak L, Marvig RL, Damkiær S, Workman CT (2011). Evolutionary dynamics of bacteria in a human host environment. Proc Natl Acad Sci USA.

[10] Taylor RF, Hodson ME, Pitt TL (1992). Auxotrophy of *Pseudomonas aeruginosa* in cystic fibrosis. FEMS Microbiol Lett.

[11] Barth AL, Pitt TL (1995). Auxotrophic variants of *Pseudomonas aeruginosa* are selected from prototrophic wild-type strains in respiratory infections in patients with cystic fibrosis. J Clin Microbiol.

[12] Barth AL, Woodford N, Pitt TL (1998). Complementation of methionine auxotrophs of *Pseudomonas aeruginosa* from cystic fibrosis. Curr Microbiol.

[13] McGill SL, Yung Y, Hunt KA, Henson MA, Hanley L (2021). *Pseudomonas aeruginosa* reverse diauxie is a multidimensional, optimized, resource utilization strategy. Sci Rep.

[14] La Rosa R, Johansen HK, Molin S (2019). Adapting to the airways: metabolic requirements of *Pseudomonas aeruginosa* during the Infection of cystic fibrosis patients. Metabolites.

[15] Everett J, Turner K, Cai Q, Gordon V, Whiteley M (2017). Arginine is a critical substrate for the pathogenesis of *Pseudomonas aeruginosa* in burn wound infections. mBio.

[16] Eisha S, Morris AJ, Martin I, Yau YCW, Grasemann H (2024). Effect of l-arginine on cystic fibrosis *Pseudomonas aeruginosa* biofilms. Antimicrob Agents Chemother.

[17] Fernández-Murga ML, Rubio V (2008). Basis of arginine sensitivity of microbial *N*-acetyl-l-glutamate kinases: mutagenesis and protein engineering study with the *Pseudomonas aeruginosa* and *Escherichia coli* enzymes. J Bacteriol.

[18] Haas D, Holloway BW, Schamböck A, Leisinger T (1977). The genetic organization of arginine biosynthesis in *Pseudomonas aeruginosa*. Mol Gen Genet.

[19] Jacobs MA, Alwood A, Thaipisuttikul I, Spencer D, Haugen E (2003). Comprehensive transposon mutant library of *Pseudomonas aeruginosa*. Proc Natl Acad Sci USA.

[20] Rojas Murcia N, Lee X, Waridel P, Maspoli A, Imker HJ (2015). The *Pseudomonas aeruginosa* antimetabolite L -2-amino-4-methoxy-trans-3-butenoic acid (AMB) is made from glutamate and two alanine residues *via* a thiotemplate-linked tripeptide precursor. Front Microbiol.

[21] Lu S-E, Soule JD, Gross DC (2003). Characterization of the *argA* gene required for arginine biosynthesis and syringomycin production by *Pseudomonas syringae* pv. syringae. Appl Environ Microbiol.

[22] Haas D, Kurer V, Leisinger T (1972). N-acetylglutamate synthetase of *Pseudomonas aeruginosa*. An assay *in vitro* and feedback inhibition by arginine. Eur J Biochem.

[23] Bolger AM, Lohse M, Usadel B (2014). Trimmomatic: a flexible trimmer for illumina sequence data. Bioinformatics.

[24] Bankevich A, Nurk S, Antipov D, Gurevich AA, Dvorkin M (2012). SPAdes: a new genome assembly algorithm and its applications to single-cell sequencing. J Comput Biol.

[25] Seemann T (2014). Prokka: rapid prokaryotic genome annotation. Bioinformatics.

[26] Darling ACE, Mau B, Blattner FR, Perna NT (2004). Mauve: multiple alignment of conserved genomic sequence with rearrangements. Genome Res.

[27] Li H, Handsaker B, Wysoker A, Fennell T, Ruan J (2009). 1000 genome project data processing subgroup. (2009). The sequence alignment/map format and samtools. Bioinformatics.

[28] Page AJ, Taylor B, Delaney AJ, Soares J, Seemann T (2016). *SNP-sites*: rapid efficient extraction of SNPs from multi-FASTA alignments. Microb Genom.

[29] Wang M, Medarametla P, Kronenberger T, Deingruber T, Brear P (2025). *Pseudomonas aeruginosa* acyl-CoA dehydrogenases and structure-guided inversion of their substrate specificity. Nat Commun.

[30] Smyth G (2005). Bioinformatics and Computational Biology Solutions Using R and Bioconductor. (Eds. Robert Gentleman et Al.).

[31] Behrends V, Tredwell GD, Bundy JG (2011). A software complement to AMDIS for processing GC-MS metabolomic data. Anal Biochem.

[32] Berkowitz DB, Charette BD, Karukurichi KR, McFadden JM (2006). α-vinylic amino acids: occurrence, asymmetric synthesis and biochemical mechanisms. Tetrahedron Asymmetry.

[33] Lee X, Fox Á, Sufrin J, Henry H, Majcherczyk P (2010). Identification of the biosynthetic gene cluster for the *Pseudomonas aeruginosa* antimetabolite l-2-amino-4-methoxy-trans-3-butenoic acid. J Bacteriol.

[34] Nandineni MR, Gowrishankar J (2004). Evidence for an arginine exporter encoded by *yggA* (*argO*) that is regulated by the LysR-type transcriptional regulator ArgP in *Escherichia coli*. J Bacteriol.

[35] Askenasy I, Swain JEV, Ho P-M, Nazeer RR, Welch A (2024). Wild type. Microbiology.

[36] Choi Y, Chan AP (2015). PROVEAN web server: a tool to predict the functional effect of amino acid substitutions and indels. Bioinformatics.

[37] Monson R, Foulds I, Foweraker J, Welch M, Salmond GPC (2011). The *Pseudomonas aeruginosa* generalized transducing phage phiPA3 is a new member of the phiKZ-like group of “jumbo” phages, and infects model laboratory strains and clinical isolates from cystic fibrosis patients. Microbiology.

[38] Lee J, Wu J, Deng Y, Wang J, Wang C (2013). A cell-cell communication signal integrates quorum sensing and stress response. Nat Chem Biol.

[39] Giegé R, Eriani G (2023). The tRNA identity landscape for aminoacylation and beyond. Nucleic Acids Res.

[40] Lu C-D, Yang Z, Li W (2004). Transcriptome analysis of the ArgR regulon in *Pseudomonas aeruginosa*. J Bacteriol.

[41] Benkert B, Quäck N, Schreiber K, Jaensch L, Jahn D (2008). Nitrate-responsive NarX-NarL represses arginine-mediated induction of the *Pseudomonas aeruginosa* arginine fermentation arcDABC operon. Microbiology.

[42] Sancho-Vaello E, Fernández-Murga ML, Rubio V (2008). Site-directed mutagenesis studies of acetylglutamate synthase delineate the site for the arginine inhibitor. FEBS Lett.

[43] Sancho-Vaello E, Fernández-Murga ML, Rubio V (2012). Functional dissection of N-acetylglutamate synthase (ArgA) of *Pseudomonas aeruginosa* and restoration of its ancestral *N*-acetylglutamate kinase activity. J Bacteriol.

[44] Sakamoto A, Sahara J, Kawai G, Yamamoto K, Ishihama A (2020). Cytotoxic mechanism of excess polyamines functions through translational repression of specific proteins encoded by polyamine modulon. Int J Mol Sci.

[45] Haas D, Leisinger T (1974). Multiple control of *N*-acetylglutamate synthetase from *Pseudomonas aeruginosa*: synergistic inhibition by acetylglutamate and polyamines. Biochem Biophys Res Commun.

