# Supplementary material for: An extragenic fix for a broken enzyme
Source: Microbiology (Reading). 2026 Oct 5;172(10):001760. doi: 10.1099/mic.0.001760 (PMC13637947; doi:10.1099/mic.0.001760)
Supplement: Supplementary file 1 [file mic-172-01760-s001.pdf]

Supplementary Information

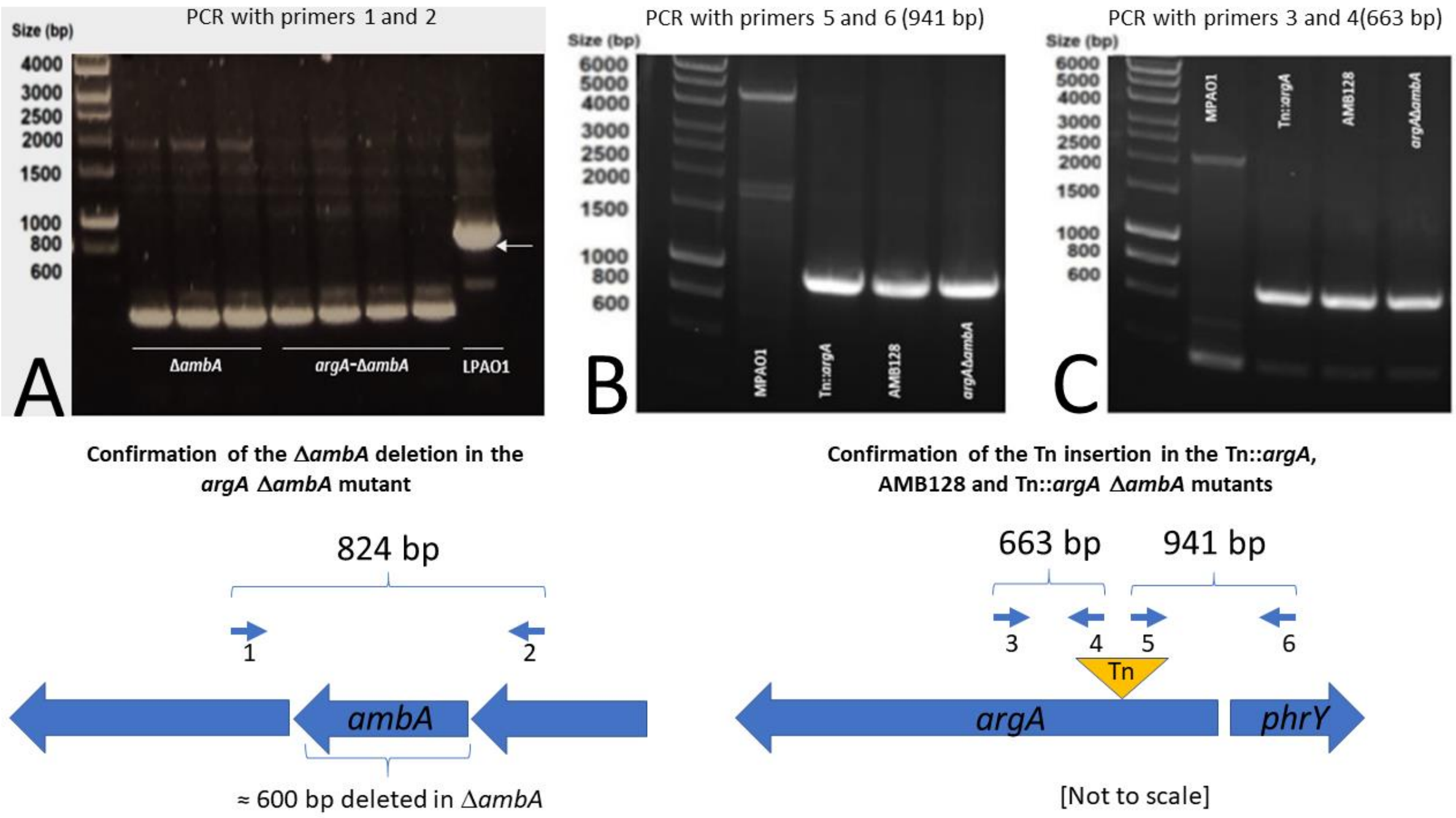

Figure S1. (A) **PCR confirmation of the in-frame deletion in Δ*ambA* and in the double mutant (Tn::*argA* Δ*ambA*).** Primers annealing at the indicated positions flanking the *ambA* ORF were used to confirmed the deletion in *ambA*. For the wild type progenitor, LPAO1, the expected band size was 824 bp. A smaller band size was expected for *ΔambA* and the Tn::*argA* Δ*ambA* mutant due to the in-frame deletion in *ambA*. (B,C) **PCR confirmation of the Tn insertion in the Tn::*argA* mutant, AMB128 (the Tn::*argA* *ambA*^{A128V} double mutant), and the Tn::*argA* Δ*ambA* double mutant.** (B) Primer pairs 5 and 6 (which anneal at the indicated positions, relative to the Tn insertion site) were expected to yield a band size of 941 bp by amplifying across the junction between the upstream region of *argA* and the beginning of the Tn insertion. A band of the correct size was observed in all of the indicated strains except MPAO1. (C) Primer pairs 3 and 4 were expected to yield band size of 663 bp by amplifying across the junction between the downstream region of *argA* and the end of the Tn insertion. Again, a band of the expected size was evident for all strains except MPAO1.

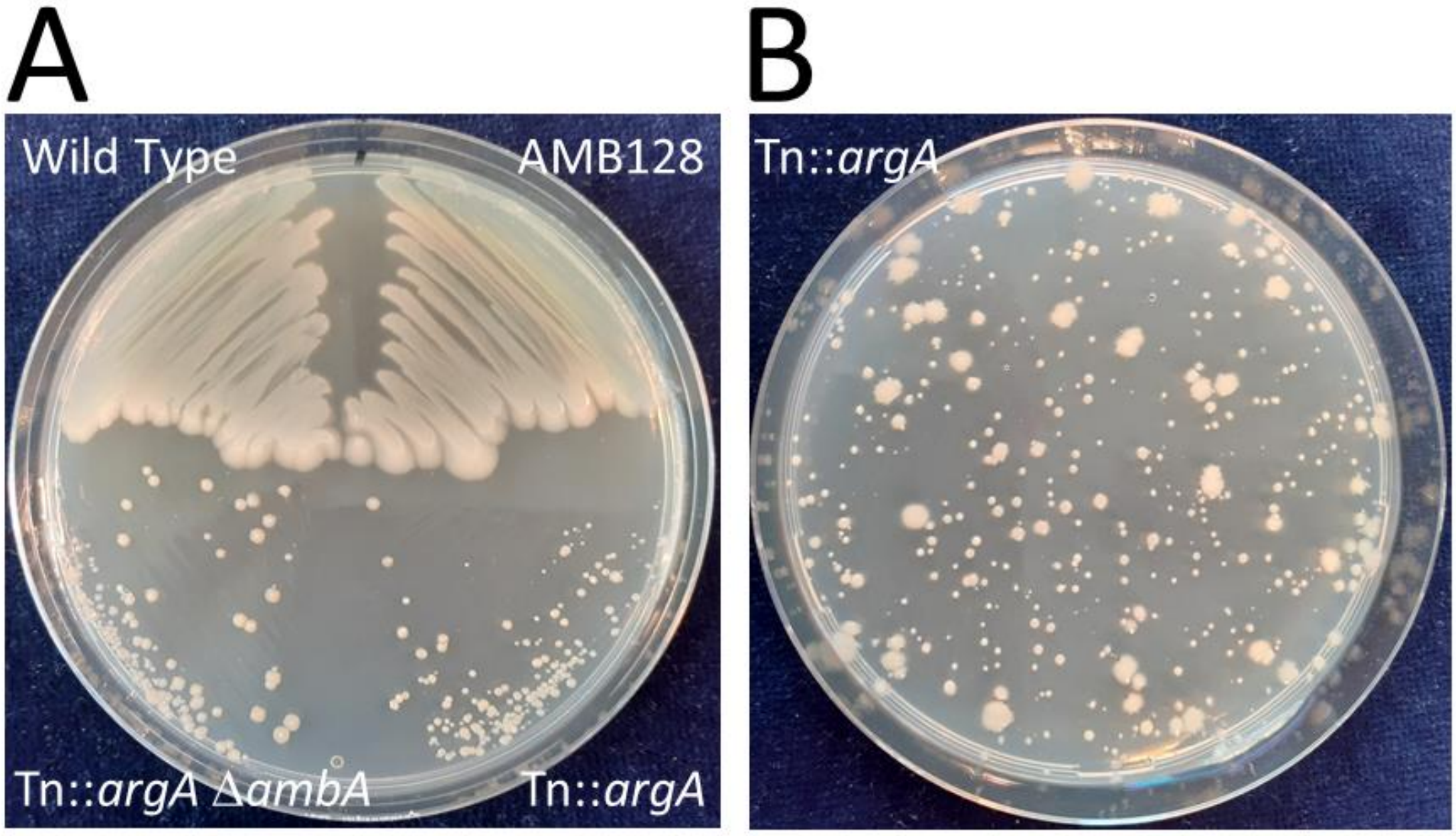

Figure S2. **Spontaneous prototrophs appear in streaks (A) and a spread (B) of the Tn::*argA* mutant (PW9762)**. The figure shows minimal medium agar plates struck with LB-grown cultures of the indicated strains in the left hand panel, or plated with 100 μL (corresponding to 1.3×10^7 CFU) of a 10 × diluted overnight culture of the Tn::*argA* mutant on the right hand panel. The overnight LB-grown cultures were washed three times in minimal medium before plating. Note the appearance of spontaneous arginine prototrophs in the Tn::*argA* mutant and the Tn::*argA* Δ*ambA* mutant in the left panel, and the heterogeneity in prototroph colony morphology/size in the right panel. Prototrophic variants appeared after 3 days incubation at room temperature.

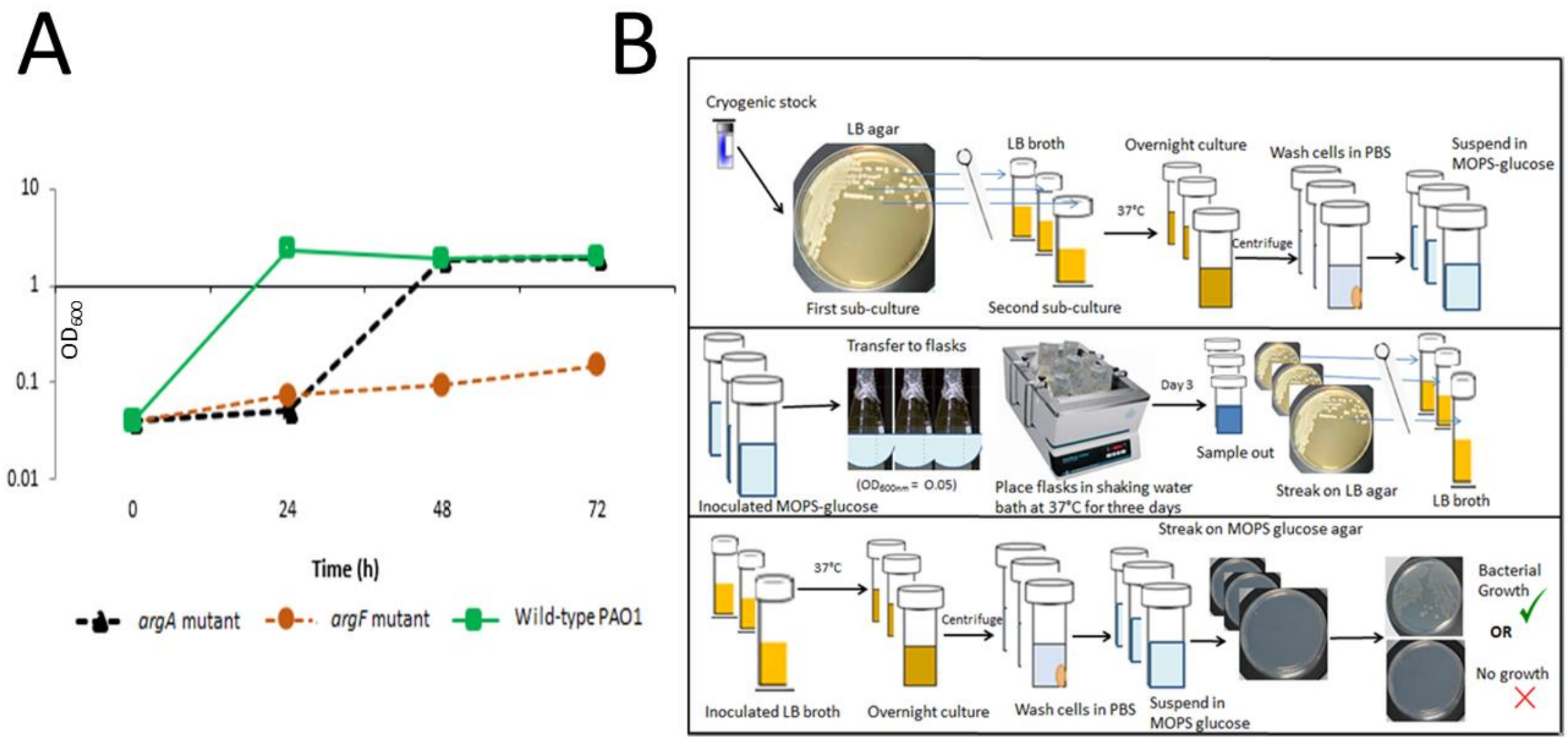

Figure S3. **(A) Growth of PW9762 (Tn::*argA* ■), PW6991 (Tn::*argF* ●), and PAO1 (■) in MOPS-glucose minimal medium, measured over 72 h**. Cells collected from overnight cultures grown in LB were washed in sterile PBS and re-suspended in MOPS-glucose minimal medium and diluted to an initial OD_{600} of 0.05. The cultures were grown in flasks in a shaking water bath (210 rpm) at 37°C for 72 h. The optical density of the cultures was measured at 600 nm, and values are expressed as mean ± SD, based on six independent biological replicates. **(B) Growth curve and *in vitro* serial passage experimental design**. Mutants and wild type were plated onto LBA from frozen stocks and grown overnight. Three separate bacterial colonies were selected and grown overnight in 10 mL of LB at 37°C. These overnight cultures were pelleted and washed twice in sterile PBS before being re-suspended in MOPS-glucose minimal medium. These samples were used to inoculate (separately) flasks containing 200 mL MOPS-glucose an initial OD_{600} of 0.05. The cultures were then grown in a shaking water bath at 37°C with good aeration (210 rpm) for three days. After this, aliquots from each flask were streaked to single colonies on LB-agar plates. Single colonies from this step were then grown up in 10 mL LB and finally, washed in PBS. The washed cells were suspended in MOPS-glucose medium and plated on MOPS-glucose minimal agar without supplemented arginine. The plates were incubated at 37°C overnight and observed for the growth of possible secondary ("bypass") mutants.

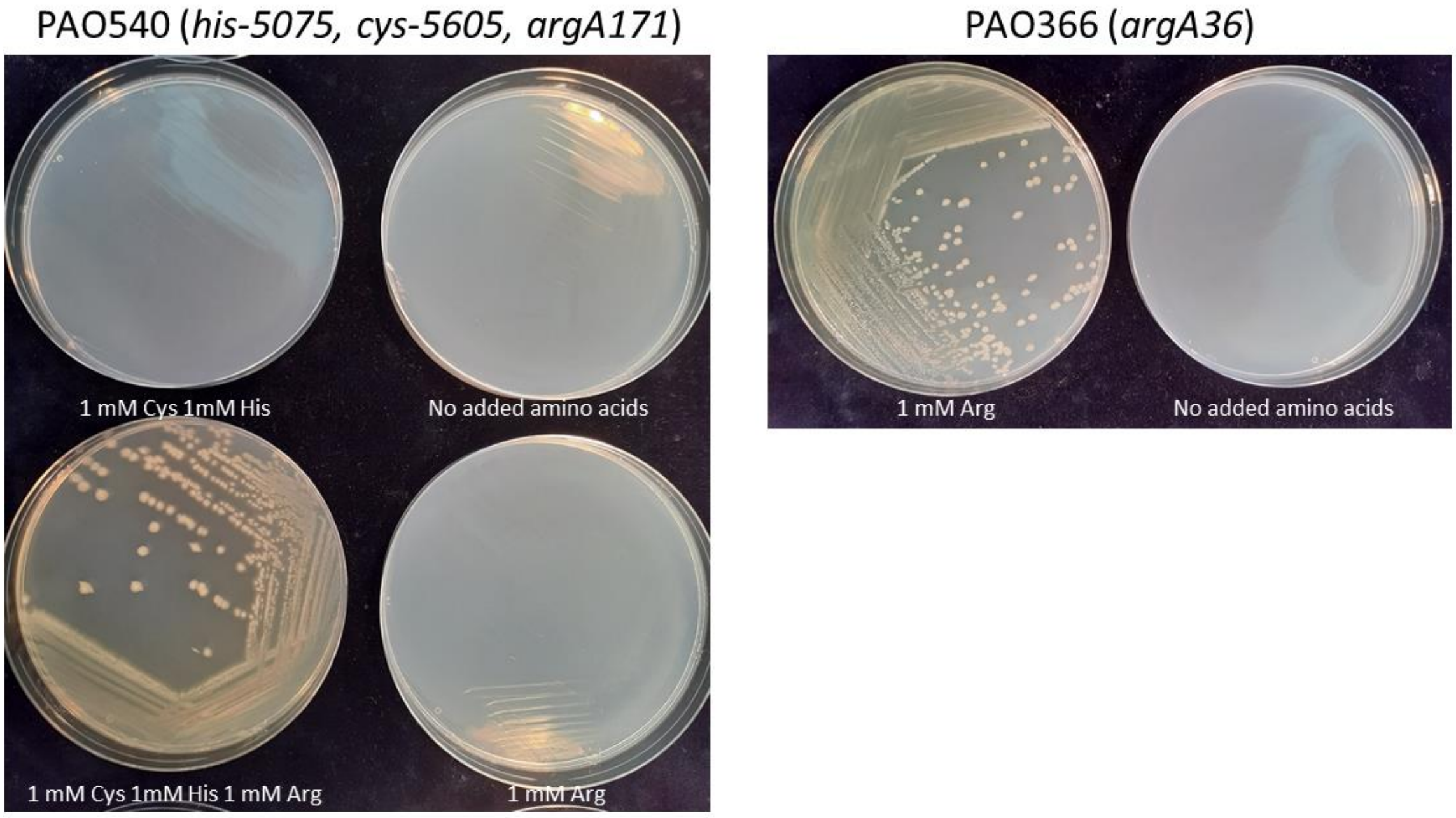

Figure S4. **Arginine auxotrophy of the PAO540 and PAO366 *argA* mutants originally isolated by Dieter Haas**. The figure shows minimal media agar plates containing the indicated additives and struck with PAO540 (left panel) and PAO366 (right panel). The plates were incubated for 24 hours at 37°C, followed by 24 hours at room temperature before photographing. Note that PAO540 manifests histidine and cysteine auxotrophy in addition to arginine auxotrophy.

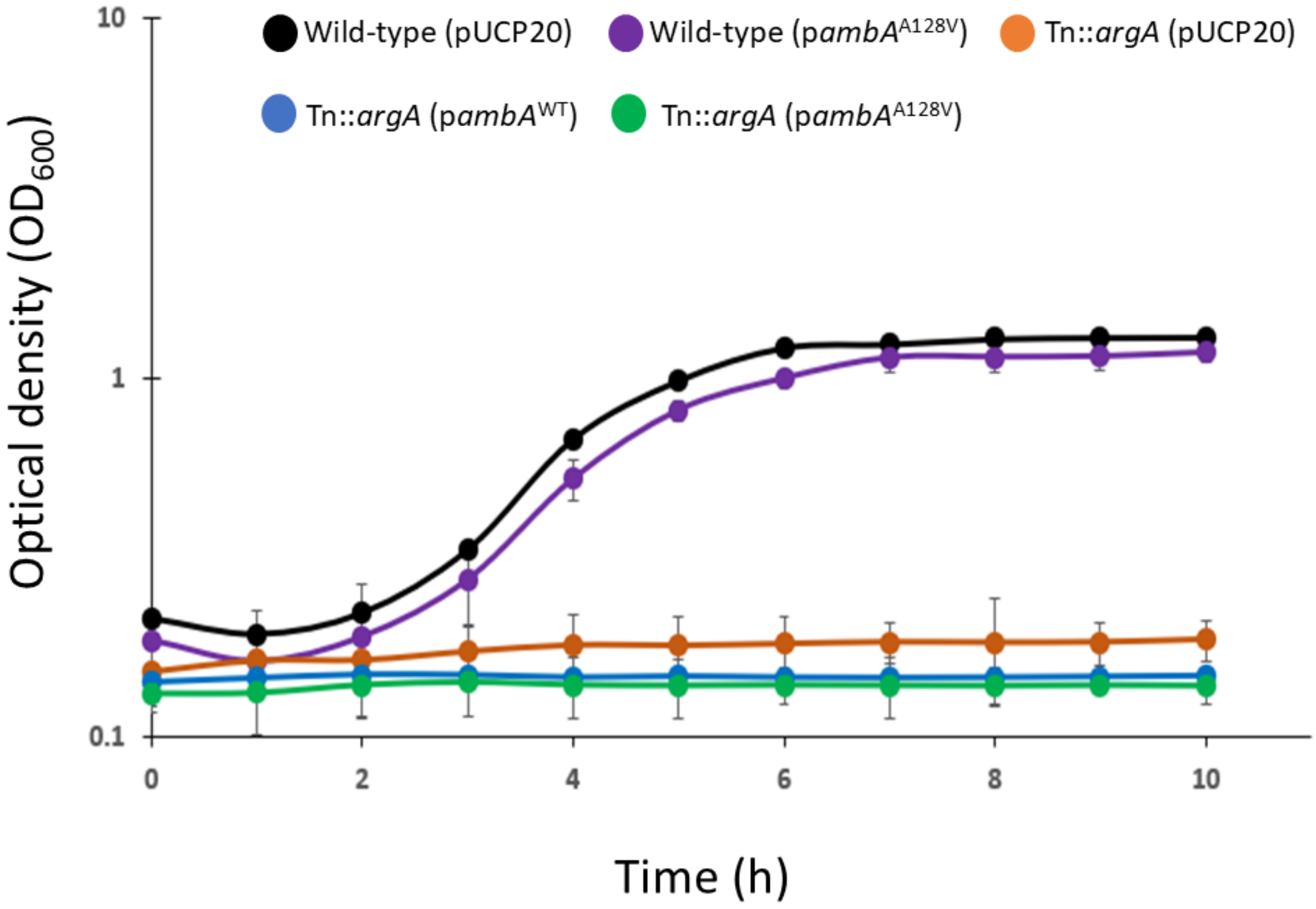

Figure S5. **Growth of the Tn::*argA* mutant and PAO1 complemented with plasmid-borne *ambA* from the wild type progenitor or from the AMB128 mutant.** The indicated strains (either "wild type" PAO1, or the Tn::*argA* mutant) contained the plasmid shown in brackets. The cultures were grown in MOPS-glucose minimal medium with good aeration at 37ºC. The OD_{600} of the cultures was measured hourly and the results are expressed as the mean ± SD for N = 3 independent biological replicates. The plasmids pUCP20, p*ambA* and p*ambA*^{A128V} represent the empty pUCP20 vector, recombinant pUCP20 vector carrying *ambA* cloned from PAO1, and recombinant pUCP20 vector with *ambA* cloned from AMB128, respectively.

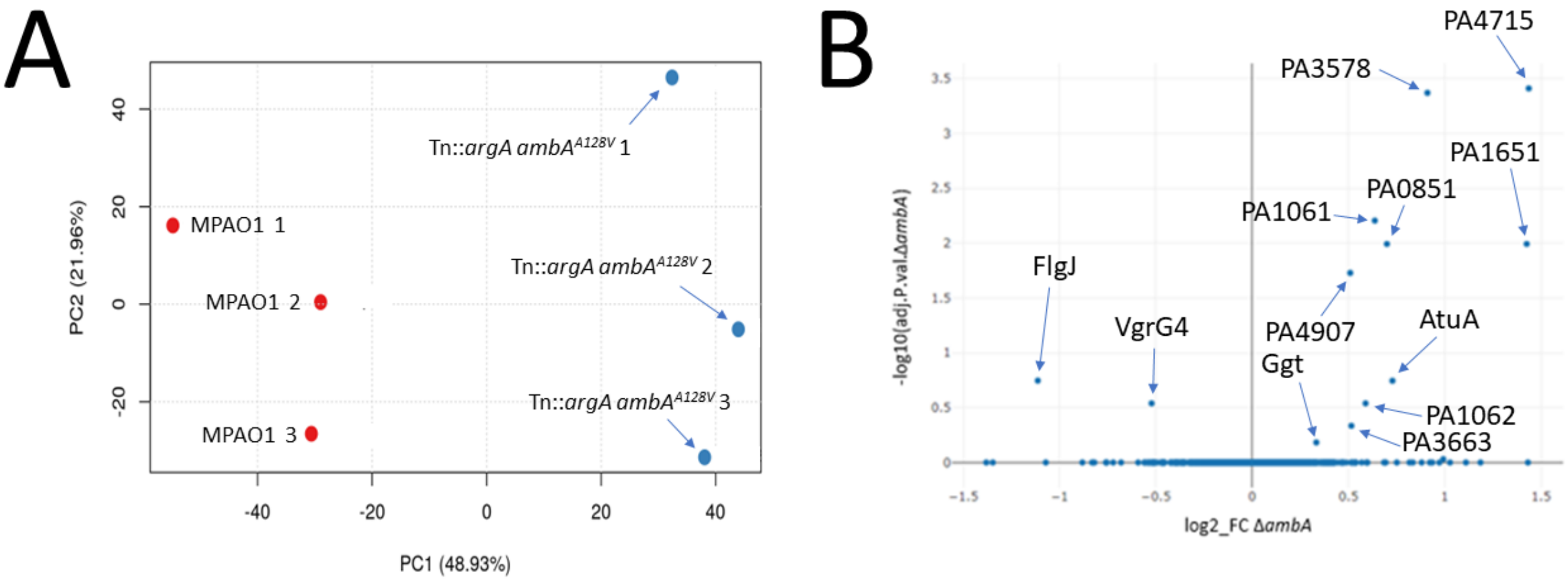

Figure S6. (A) **Principal Component(s) Analysis (PCA) scores plot** illustrating the separation (primarily along PC1) between the proteome associated with MPAO1 and AMB128 (the Tn::*argA ambA*^{A128V} mutant). N = 3 independent biological replicates each of MPAO1 and AMB128 were analyzed. (B) **Volcano plot** comparing the intracellular proteome of the Δ*ambA* mutant (N = 3 replicates) with that of its progenitor, LPAO1 (N = 2 replicates). Interestingly, AmbA was not among the modulated proteins in the LPAO1 samples, suggesting that the protein is likely to be expressed at very low levels.

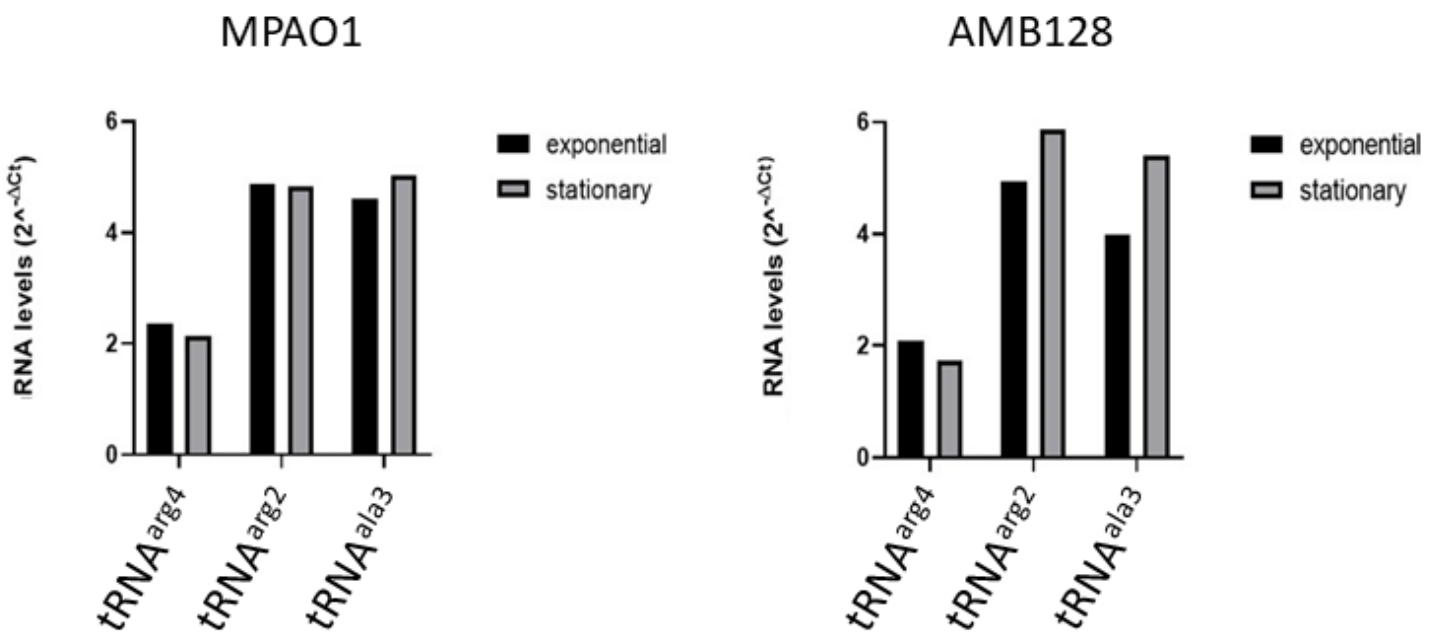

Oligo name	Sequence (5’-3’)
tRNA^{arg4} fwd	GGTTCGATAGCTCAGCCTGG
tRNA^{arg4} rev	TTCGAGCGGGAGTCGAAC
tRNA^{arg3} fwd	GCTCAGCTGGATAGAGTACTG
tRNA^{arg3} rev	CATCCGGCGGGATTCGAAC
tRNA^{ala3} fwd	GGCCATAGCTCAGCTGGGAG
tRNA^{ala3} rev	GTGGAGCCAAGGAGGATCG
rpoD fwd	CTGAAGATCGCCAAAGAGCC
rpoD rev	GTGTGGTCGGTGTTCATGTC

Figure S7. **Expression levels of tRNA^{arg4} (PA4581.1), tRNA^{arg2} (PA0263.1) and tRNA^{ala3} (PA4280.3) in LB-grown cultures of MPAO1 and AMB128.** Total RNA was extracted from 2 mL samples of MPAO1 or AMB128 cultures grown in LB with good aeration to the exponential phase (OD_{600} = 0.6–0.8) or stationary phase (OD_{600} = 1.8–2.0) of growth, as indicated. Cells were harvested by centrifugation, resuspended in Max Bacterial Enhancement Reagent, and boiled at 95 °C for 10 min. Trizol (Qiagen) and cold chloroform were then added to lyse the cells and induce phase separation. The samples were then centrifuged to partition the RNA into the upper aqueous phase. RNA from the upper aqueous phase was purified using the Monarch Spin RNA Cleanup Kit (NEB) according to the manufacturer’s instructions. cDNA was synthesized from 1 µg of total purified RNA using SuperScript III Reverse Transcriptase (Invitrogen) according to the manufacturer’s instructions. qRT-PCR was performed in triplicate using Luna® Universal qPCR Master Mix (NEB) using the indicated forward and reverse primers in the Table on a QuantStudio Real-Time PCR System (Thermo Fisher). Relative gene expression for each strain was calculated by normalizing the tRNA levels to *rpoD* mRNA (ΔCt), ensuring consistent control Ct values across strains and conditions for accurate normalization. tRNA levels are displayed graphically as $2^{-\Delta Ct}$ values.

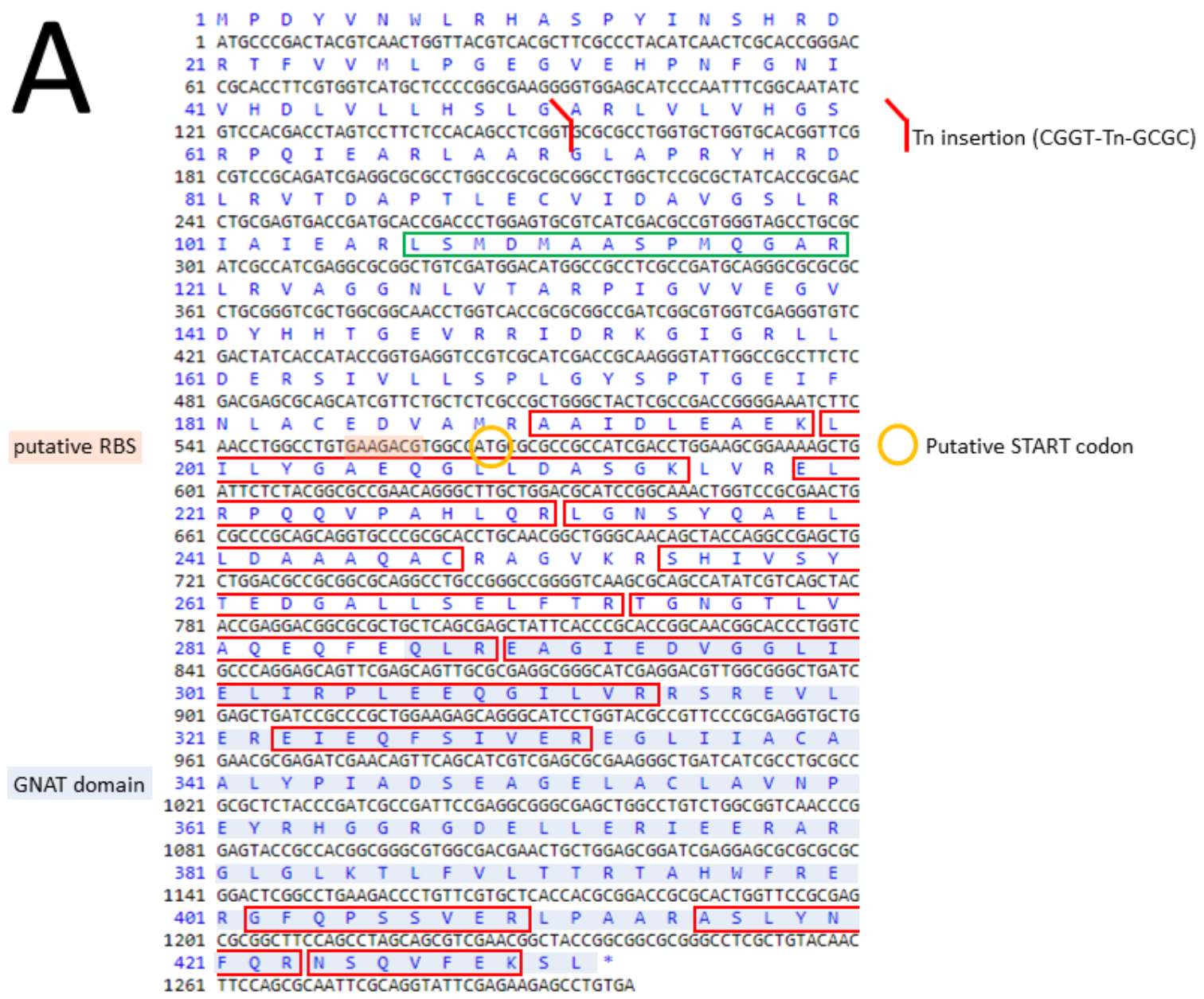

B

Relative quantitation of peptides

Sequence	MPAO1	AMB128	LPAO1	*ΔambA*
LSMDMAASPMQGAR	36.2	1.8	67.1	52.9
AAIDLEAEK	96.8	239.0	101.5	101.1
LILYGAEQGLLDASGK	105.0	272.6	133.6	129.9
ELRPQQVPAHLQR	46.5	133.3	48.5	50.7
LGNSYQAELLDAAAQAC	20.6	56.3	25.0	21.6
SHIVSYTEDGALLSELFTR	54.8	148.8	79.7	57.8
TGNGTLVAQEQFEQLR	50.0	151.1	51.3	56.7
EAGIEDVGGLIELIRPLEE(	3.6	16.5	3.9	4.3
EIEQFSIVER	30.3	104.2	28.8	26.0
GFQPSSVER	87.3	293.8	77.8	87.7
ASLYNFQR	39.1	71.9	62.7	57.9
NSQVFEK	110.6	336.5	91.3	97.7

Figure S8. **Mapping and quantitation of ArgA-derived peptides in MPAO1 and AMB128**. (A) **Nucleotide and amino acid sequence of ArgA** showing the location of the Tn insertion in PW9762 (and in AMB128) and the location of tryptic peptides associated with ArgA from the MPAO1, AMB128, LPAO1, and Δ*ambA* proteomic samples. Peptides highlighted in red were identified in all of the strains examined, whereas the peptide highlighted in green was barely detectable in AMB128 but was abundant in the other samples. The putative alternative start codon (Met188) in AMB128 is highlighted, as is the preceding RBS. The GNAT domain is shaded as indicated. (B) **The table shows the relative quantitation of each indicated peptide** in the samples from MPAO1, AMB128, LPAO1, and the Δ*ambA* mutant.

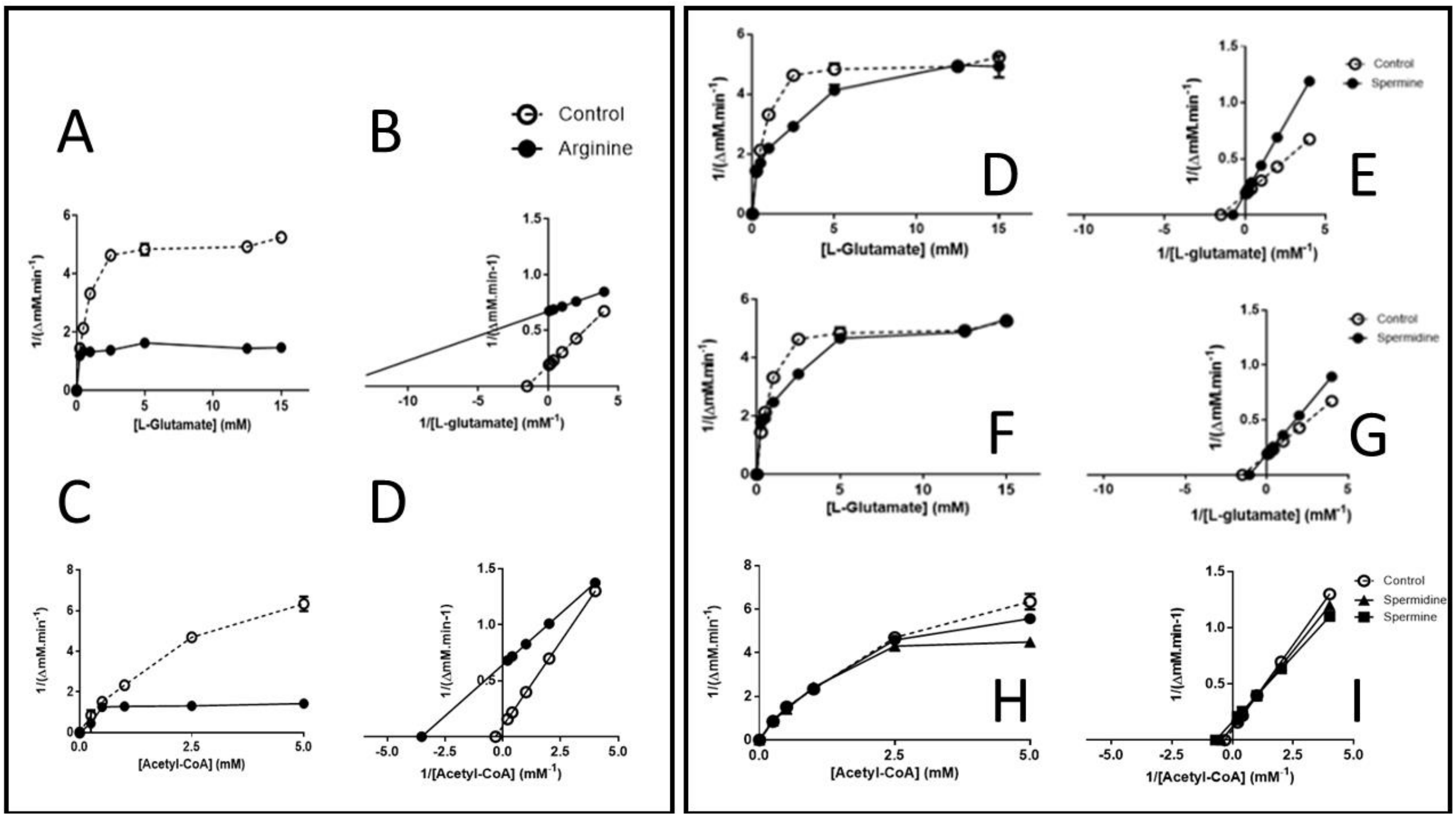

Figure S9. **Arginine is an uncompetitive inhibitor of ArgA whereas polyamines are competitive inhibitors of the enzyme.** The left panel (A-D) shows kinetic data (Michaelis-Menten plots (A,C) and associated Lineweaver-Burk plots (B,D) showing the effect of arginine (1 mM) on the NAGS activity of full-length ArgA in the presence of varying concentrations of glutamate (A,B; $[acetyl\text{-}CoA]_{initial}$ fixed at 3 mM) and acetyl-CoA (C,D; $[glutamate]_{initial}$ fixed at 15 mM) substrates. The right panel (E-I) contains Michaelis-Menten (D,F,H) and Lineweaver-Burk (E,G,I) plots showing the effect of the polyamines, spermine (1 mM, D,E) and spermidine (1 mM, F,G) on the glutamate dependency of ArgA ($[acetyl\text{-}CoA]_{initial}$ = 3 mM). Plots H and I show the dependency of ArgA NAGS activity on [acetyl-CoA] in the absence of polyamine or in the presence of spermine or spermidine (both 1 mM). Values represent the mean of three replicates ± standard deviation. Figures were generated using GraphPad Prism7.

Table S1. List of the top 20 most up-regulated proteins associated with AMB128 (the *ambA*^{A128V} Tn::*argA* mutant) *cf*. MPAO1. Modulations of the same proteins in the ∆*ambA* mutant *cf*. LPAO1 are shown for comparison. All measurements represent the mean from N = 3 biological replicates except LPAO1, where there were only 2 biological replicates. Note that there is essentially no overlap between the proteins modulated in AMB128 and the ∆*ambA* mutant. Also note that ArgA (encoding the NAGS activity) is the 20th most highly up-modulated protein and is shown in bold red text. The locus tags of ORFs likely associated with common operons are shaded in block colours.

**Locus tag**	**gene description**	**AMB128**		***ΔambA***	
		log_2_FC	adj.P.Val	log_2_FC	adj.P.Val
**PA4582**	Uncharacterized (*rtc* cluster)	3.2	4.3E-05	0.834	0.999
**PA1844**	*tse1*	3.0	2.57E-05	-0.001	0.999
**PA2702**	*tse2*	2.9	7.05E-05	-0.036	0.999
**PA4584**	Uncharacterized (*rtc* cluster)	2.8	5.12E-05	0.464	0.999
**PA0277**	M48 family peptidase (Zn^{2+}-dependent)	2.8	0.000111	-0.072	0.999
**PA3385**	Alginate and motility regulator Z (*amrZ*)	2.8	2.94E-05	-0.171	0.999
**PA4585**	RNA 3'-terminal phosphate cyclase (*rtcA*)	2.7	2.57E-06	-0.007	0.999
**PA5114**	Uncharacterized transmembrane protein	2.4	1.27E-05	0.081	0.999
**PA4583**	Uncharacterized (probable *rtcB*)	2.3	6.65E-06	0.037	0.999
**PA0046**	Uncharacterized (TPR repeat protein)	2.3	3.83E-05	-0.144	0.999
**PA3729**	Uncharacterized coil-rich protein	2.2	1.05E-05	0.054	0.999
**PA0047**	Uncharacterized probable lipoprotein	2.1	1.05E-05	-0.350	0.999
**PA3730**	Uncharacterized protein adjacent to PA3729 (above)	2.0	9.77E-06	-0.067	0.999
**PA5113**	Uncharacterized	2.0	7.15E-06	0.024	0.999
**PA4624**	cyclic diguanylate-regulated TPS partner B (*cdrB*)	1.9	1E-05	-0.111	0.999
**PA3727**	Uncharacterized PLD-like protein	1.9	6.49E-06	-0.170	0.999
**PA2040**	Glutamylpolyamine synthetase *pauA4*	1.9	6.25E-05	-0.004	0.999
**PA5134**	C-terminal processing protease *ctpA*	1.8	2.57E-06	0.055	0.999
**PA5107**	Outer membrane lipoprotein	1.8	6.43E-05	0.084	0.999
**PA5204**	**N-acetylglutamate synthase (*argA*)**	**1.8**	**3.83E-05**	**0.017**	**0.999**

Table S2. List of down-regulated proteins associated with AMB128 (the *ambA*^{A128V} mutant) *cf*. MPAO1, and the ∆*ambA* mutant *cf*. LPAO1. All measurements represent the mean from N = 3 biological replicates except LPAO1, where there were only 2 biological replicates. Note that there is essentially no overlap between the proteins modulated in AMB128 and the ∆*ambA* mutant. The locus tags of ORFs likely associated with the same or functionally linked operons are shaded in block colours.

		AMB128		Δ*ambA*	
**Locus tag**	**gene description**	log_2_FC	adj.P.Val	log_2_FC	adj.P.Val
**PA1821**	Probable enoyl-CoA hydratase/isomerase	-2.603	6.6E-07	-0.01977	1.000
**PA1780**	*nirD*	-2.114	2.06E-05	-0.00807	1.000
**PA3880**	Uncharacterized diguanylate cylase	-2.092	3.4E-05	-0.14748	1.000
**PA0525**	*norD*	-2.000	7.62E-06	0.08090	1.000
**PA0520**	*nirQ*	-1.842	2.49E-05	0.11292	1.000
**PA3873**	*narJ*	-1.722	1.3E-05	0.03846	1.000
**PA4502**	Probable dipeptide binding protein component of ABC transporter (*dppA4*)	-1.668	2.57E-06	-0.11802	1.000
**PA3688**	Uncharacterized	-1.656	5.16E-05	-0.15487	1.000
**PA2381**	Uncharacterized orphan bacteriophage protein	-1.650	3.2E-05	-0.12184	1.000
**PA4500**	Probable dipeptide binding protein component of ABC transporter (*dppA3*)	-1.635	8.51E-06	-0.19089	1.000
**PA3913**	Probable protease	-1.478	6.89E-05	-0.02669	1.000
**PA5429**	Aspartate ammonia-lyase (*aspA*)	-1.473	3.88E-05	-0.08709	1.000
**PA1894**	Probable αKG/Fe^{2+} monooxygenase	-1.380	1.05E-05	-0.23212	1.000
**PA0176**	Aerotaxis transducer (*aer2*)	-1.334	2.13E-05	-0.02259	1.000
**PA2119**	Alcohol dehydrogenase (Zn^{2+}-dependent)	-1.301	9.15E-06	-0.00175	1.000
**PA3647**	probable outer membrane protein precursor	-1.292	9.77E-06	0.10288	1.000
**PA2664**	*fhp* (nitrosative stress response protein)	-1.281	8.08E-05	0.00300	1.000
**PA3446**	Uncharacterized FMN reductase	-1.252	1.44E-05	-0.18144	1.000
**PA4587**	*ccpR*	-1.239	0.00020	0.0257	1.000
**PA4336**	Uncharacterized PfpI-like deglycase	-1.180	2.1E-05	-0.06591	1.000

**PA1096**	Probable *fliT*	-1.158	0.00045	-0.11835	1.000
**PA0175**	*cheR2*	-1.157	0.00034	-0.20942	1.000
**PA4309**	Chemotactic transducer (*pctA*)	-1.154	6.74E-05	0.138901	1.000
